# Seed Imbibition and Metabolism Contribute Differentially to Initial Assembly of the Soybean Holobiont

**DOI:** 10.1094/PBIOMES-03-23-0019-MF

**Published:** 2022-07-18

**Authors:** Davide Gerna, David Clara, Livio Antonielli, Birgit Mitter, Thomas Roach

**Affiliations:** 1 Department of Botany and Center for Molecular Biosciences Innsbruck, University of Innsbruck, 6020 Innsbruck, Austria; 2 Center for Health & Bioresources, Bioresources Unit, AIT Austrian Institute of Technology GmbH, 3430 Tulln, Austria Accepted for publication 17 August 2023

**Keywords:** abscisic acid, *Bacillus*, *Buchnera*, *Glycine max* (soybean), imbibition, metabolism, microbiome, *Rhodococcus*, seed germination

## Abstract

Seed germination critically determines successful plant establishment and agricultural productivity. In the plant holobiont’s life cycle, seeds are hubs for microbial communities’ assembly, but what exactly shapes the holobiont during germination remains unknown. Here, 16S rRNA gene amplicon sequencing characterized the bacterial communities in embryonic compartments (cotyledons and axes) and on seed coats pre- and post-germination of four soybean (*Glycine max*) cultivars, in the presence or absence of exogenous abscisic acid (ABA), which prevented germination and associated metabolism of seeds that had imbibed. Embryonic compartments were metabolically profiled during germination to design minimal media mimicking the seed endosphere for bacterial growth assays. The distinction between embryonic and seed coat bacterial microbiomes of dry seeds weakened during germination, resulting in the plumule, radicle, cotyledon, and seed coat all hosting the same most abundant and structurally influential genera in germinated seeds of every cultivar. Treatment with ABA prevented the increase of bacterial microbiomes’ richness, but not taxonomic homogenization across seed compartments. Growth assays on minimal media containing the most abundant metabolites that accumulated in germinated seeds revealed that seed reserve mobilization promoted enrichment of copiotrophic bacteria. Our data show that seed imbibition enabled distribution of seed-coat-derived epiphytes into embryos irrespective of germination, while germinative metabolism promoted proliferation of copiotrophic taxa, which predominated in germinated seeds.

Seeds are capsules of both plant and microbial life. In a so-called plant holobiont, microbiomes, defined as microbial communities acting in a certain niche with definite physicochemical characteristics (Berg et al. 2020; Whipps et al. 1988), are holistically viewed as sources of additional genes and functions for the plant host (Bordenstein and Theis 2015; Jefferson 1994; Vandenkoornhuyse et al. 2015; Zilber-Rosenberg and Rosenberg 2008). In turn, the plant host appears capable of modulating the composition of its microbiomes throughout the holobiont’s life cycle, particularly under environmental fluctuations (Bulgarelli et al. 2013; Mitter et al. 2016; Rochefort et al. 2019; Sanchez-Cañizares et al. 2017; Trivedi et al. 2020). Partnerships between plants and microbes are already established in both embryonic and nonembryonic tissues during seed development (Chesneau et al. 2020; Gerna et al. 2022; Liu et al. 2013; Nelson 2018). Seed microbial communities pioneer the primary assemblage of the adult plant’s microbiome, representing an initial microbial reservoir (Barret et al. 2015; Johnston-Monje et al. 2016; Nunes et al. 2022; Shade et al. 2017; Torres-Cortés et al. 2018; Walsh et al. 2021). Furthermore, microbial communities can influence seed germination, vigor, and early seedling growth through neutral, beneficial, and detrimental effects (Barret et al. 2016; Bergna et al. 2018; Escobar Rodríguez et al. 2020; Gerna et al. 2022; Hardoim et al. 2015; Shade et al. 2017; Simonin et al. 2022; Truyens et al. 2015; Tyc et al. 2020). External plant surfaces and internal tissues (i.e., the endosphere) can be colonized by microbes, leading to a distinction between epiphytic and endophytic communities (Brader et al. 2017; Nelson 2018).

In seeds, the endosphere comprises the embryo and nonembryonic storage tissues (e.g., endosperm or cotyledons) inside of the seed coat, and many seed endophytes originate from the soil through colonization of the rhizosphere (Escobar Rodríguez et al. 2020, 2024; Rochefort et al. 2021). Some endophytic microorganisms can be transmitted vertically from the seed to the progeny plants (Frank et al. 2017; Nelson 2018), including after flower colonization (Mitter et al. 2017), while a fraction of the endophytic pool displays heritability (i.e., transmission from seed to seed; Abdelfattah et al. 2021; Bergna et al. 2018; Escobar Rodríguez et al. 2020: Johnston-Monje and Raizada 2011; Rezki et al. 2018). Epiphytes on seed coats can have more heterogeneous origins, including fruit and flower debris at late seed maturation stages, but also air and soil through recruitment by horizontal transmission (Links et al. 2014; Lymperopoulou et al. 2016). In crops, seed harvesting, processing, and storage provide further opportunities for erratic seed colonization by epiphytes, often comprising opportunistic and potentially pathogenic species (Nelson 2018). Only a few previous culture-independent studies have drawn attention to the relevance of distinct seed compartments (seed coat, embryos, and storage tissues) to understanding shifts in the endophytic bacterial communities during seed germination (Abdelfattah et al. 2021; Kuźniar et al. 2020; Lopez-Velasco et al. 2013; Ofek et al. 2011). However, in such studies, seed surface sterilization or poor DNA quality precluded assessing the dynamics of epiphytic communities, some of whose members, similarly to endophytes, are believed to interact persistently with their hosts, thus affecting germination performance through, for instance, protection from pathogens (Links et al. 2014).

For desiccated seeds to germinate, a first requirement is water uptake, termed imbibition, which enables transcription, translation, and resumption of energetic metabolism, including mitochondrial respiration and associated glycolysis and the pentose phosphate pathway (Nonogaki et al. 2010; Rosental et al. 2014). Nutrients are also essential for the growth of plant-associated bacteria. Thus, breakdown of seed reserves from storage tissues may provide primary metabolites not only to the autotroph seedling, but also for the establishment of the holobiont. Accordingly, contrasting bacterial life strategies, reflective of growth rates in response to nutrient availability, could explain shifts in community composition during the seed-to-seedling transition (Barret et al. 2015; Ho et al. 2017; Torres-Cortés et al. 2018). Overall, studies on seeds have disclosed the relevance of ecological drivers, such as influence of parental environment, agricultural practice, selection by the plant host genotype, and ecological drift, to the assemblage of bacterial communities (Adam et al. 2018; Chen et al. 2020; Escobar Rodríguez et al. 2020; Eyre et al. 2019; Klaedtke et al. 2016; Rezki et al. 2018; Rochefort et al. 2019; Shade et al. 2017; Wassermann et al. 2022). However, the contribution of seed physiological processes, namely imbibition, resumption of seed metabolism, and mobilization of seed storage reserves, to microbiomes’ shifts across distinct seed compartments has hitherto been neglected.

Here, we hypothesized that seed imbibition and resumption of germinative metabolism each play a distinct role in assembling bacterial microbiomes. We studied seeds of the major pulse crop soybean produced from plants of four cultivars grown in different fields, including one from an organic farming regime. The native bacterial communities harbored within embryonic compartments or on seed coats were assessed with partial 16S rRNA gene amplicon sequencing, enabling determination of bacterial richness and evenness (α-diversity), alongside taxonomic structure (β-diversity). Imbibition was dissociated from seed germinative metabolism with the phytohormone abscisic acid (ABA), which prevented germination of imbibing seeds. In the cultivar most sensitive to ABA, we used gas chromatography coupled to mass spectrometry (GC-MS) to quantify primary metabolites (i.e., putative carbon sources for bacteria) during seed germination (control) and imbibition alone (i.e., with exogenous ABA). Accumulation of most primary metabolites during seed germination revealed clear positive and negative correlations to different bacterial taxa, whose abundance increased or decreased significantly, respectively. The concentrations of the most abundant metabolites detected in the endosphere of germinated seeds served to formulate minimal media to test if seed germinative metabolism promotes the assemblage of copiotophic bacteria at precocious growth stages of the plant holobiont.

## Materials and Methods

### Seed preparation for analyses

Fully mature soybean (*Glycine max* L.) seeds of the four cultivars ‘Abelina’, ‘Amadea’, ‘Amandine’, and ‘Cordoba’ were derived from plants grown in different fields in Upper Austria in 2019 under conventional farming practice, except for seeds of ‘Amandine’, which was subjected to certified EU organic cultivation practices (Supplementary Fig. S1). For simplicity, the term cultivar hereafter refers to the combined effects of plant genotype, production site, and agricultural practice. All seeds were provided by Saatbau Linz eGen (Leonding, Austria; https://www.saatbau.com/standort/saatbau-linz-egen/). Upon arrival in December 2019, seeds were handled and processed aseptically, with those damaged and immature (very small and greenish, ~1%) being discarded. Following equilibration to a water content of 0.0707 ± 0.0002 g of H_2_O g^−1^ dry weight (DW), seeds were stored at −20°C in vacuum-sealed aluminum foil laminated bags (Landig + Lava GmbH & Co. KG, Bad Saulgau, Germany) until analyzed in 2020. Seeds were not surface-sterilized before imbibition on three layers of filter paper (Whatman grade 1, GE Healthcare, Little Chalfont, U.K.) saturated with autoclaved ultrapure water (aUPW) at 20°C under constant darkness. Germination was defined as radicle protrusion through the seed coat by at least 2 mm. ABA was purchased from Sigma Aldrich (St. Louis, MO, U.S.A.) and dissolved in aUPW to the desired concentration for treatments during seed imbibition on filter paper.

**Fig. 1:**
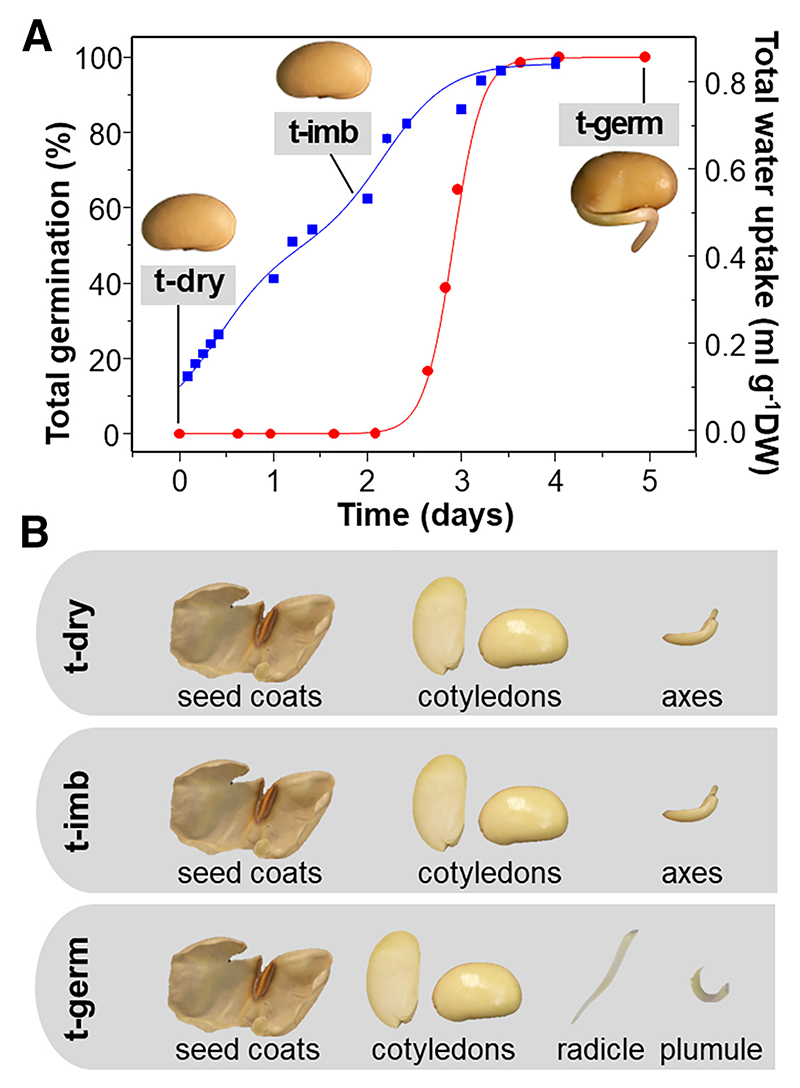
Overview of soybean germination and material analyzed. **A**, Progress of germination (red circles) and imbibition (blue squares) overlaid with images of typical seeds at time points of analysis. **B**, Images of typical seed structures analyzed at each time point. t-dry, dry seeds; t-imb, imbibed seeds just before any had germinated; t-germ, germinated seeds 5 days after imbibition started.

To analyze microbiomes (*n* = 3 replicates each of 30 seeds for each cultivar) and metabolite concentrations (*n* = 5 replicates each of 30 seeds for ‘Abelina’ only), seeds were first lyophilized for 5 days. After excision, cotyledons were ground in liquid-nitrogen-cooled MM40 25-ml stainless steel capsules with a 15-mm-diameter stainless steel ball (Retsch GmbH, Haan, Germany), and embryonic axes in liquid-nitrogen-cooled 2-ml Eppendorf tubes containing one 5-mm-diameter agate bead, using a Tissue Lyser (Qiagen GmbH, Hilden, Germany) at 30 Hz for 2 and 1.5 min, respectively. Seed coats were ground (50 Hz, 2 min) with a bead mill in 5-ml Teflon capsules with a 7-mm-diameter agate bead (Sartorius, Göttingen, Germany) precooled in liquid nitrogen. Between samples, capsules and beads were thoroughly cleaned with 100% (vol/vol) acetone, 70% (vol/vol) ethanol, and aUPW prior to auto-claving (121°C, 20 min) and 30 min exposure to UV light under a sterile flow bench. For microbiome and metabolite analyses during imbibition and germination, seeds were incubated at 20°C in the dark for 50 h and 5 days, respectively (Fig. 1). Before excision of embryonic axes from cotyledons, seed coats were peeled off aseptically at both intervals. After 5 days, germinated seeds developed a radicle at least 1 cm long, which was cut away from the nonemerged greenish plumule after excision of the seedling axis from the cotyledons (Fig. 1). Separated seed compartments were lyophilized and ground as described earlier. After being ground, samples were hermetically sealed in plastic boxes over silica gel and stored at –80°C until analysis.

### Microbiome analyses

#### Extraction of genomic DNA

Genomic DNA was extracted using approximately 150 mg of finely ground lyophilized powder of each seed compartment and a DNeasy PowerSoil Pro kit (Qiagen GmbH, Hilden, Germany), following the manufacturer’s instructions, and bead beating on a FastPrep homogenizer for cell lysis (speed setting 6, 40 s). DNA samples were eluted in 50 μl of C6 buffer, and 5-μl aliquots were loaded onto 1% (wt/vol) agarose gels in TBE (Tris-borate-EDTA) buffer for staining with Midori Green (Bulldog Bio, Portsmouth, England) and visual inspection of quality after electrophoresis (100 V, 40 min). Furthermore, DNA concentration and purity were determined using a NanoDrop ND-1000 spectrophotometer (Thermo Scientific, Waltman, MA, U.S.A.).

#### 16S rRNA gene fragment amplicon library preparation and sequencing

PCR amplifications between the V5 and V7 hypervariable regions of the 16S rRNA gene were conducted using a KAPA HiFi HotStart PCR Kit (Roche Holding AG, Basel, Switzerland) containing the high-fidelity polymerase KAPA with proofreading activity (Kapa Biosystems, Boston, MA, U.S.A.) and the Illumina-tagged primer pair 799f_ill (forward, 5′-TCGTCGGCAGCGT CAGATGTGTATAAGAGACAGAACMGGATTAGATACCCKG-3′) and 1175r_ill (reverse, 5′-GTCTCGTGGGCTCGGAGATGTGTATAAGAGACAGACGTCRTCCCCDCCTTCCTC-3′; Chelius and Triplett 2001). PCR mixtures contained 1 μl of template DNA, 1× KAPA HiFi, and 1× GC buffer (1:1 ratio) with 2 mM MgCl_2_, 300 μM dNTPs, 300 nM of each primer, 0.5 units of KAPA HiFi polymerase, and PCR-grade H_2_O up to 25 μl. Amplification conditions consisted of an initial denaturation step at 95°C for 3 min, followed by 30 cycles including denaturation at 98°C for 30 s, annealing at 55°C for 30 s, and elongation at 72°C for 30 s, and completed by a final elongation step at 72°C for 5 min. For each sample replicate, PCR amplification was repeated three times independently before amplicons were pooled together, loaded in each second well to avoid cross-contamination, and separated on 1.5% (wt/vol) agarose gels in 1× TAE buffer (Tris-acetate-EDTA, Carl Roth GmbH+Co, Karlsruhe, Germany) at 100 V for 1 h. To separate the bacterial 16S rRNA gene amplicons from amplified plant mitochondrial 18S rRNA gene, agarose gels were exposed to blue light (400 nm) from a transilluminator (MaestroGen, Hsinchu City, Taiwan), and PCR bands of 443 bp (i.e., 16S rRNA gene amplicons) were excised using Xtracta Generation II punchers (Biozym Biotech Trading, Vienna, Austria). DNA in the gel pieces was eluted by centrifugation (14,000 rpm, 3 min) through sterile 1-ml filter tips (Biozym Biotech Trading), and eluates containing 16S rRNA gene amplicons were used as templates in indexing PCR. Particularly, 1-μl aliquots of eluates were mixed with 1× KAPA HiFi buffer with 2 mM MgCl_2_, 300 μM dNTPs, 300 nM of each forward (S502-S503, S505-S508, S510-S511) and reverse (N701-N707, N710-N712, N714-N715) Nextera XT indexing primer (Illumina, San Diego, CA, U.S.A.), 0.25 U of KAPA HiFi polymerase, and PCR-grade water up to 50 μl.

Indexing PCR amplification started with a denaturation step at 95°C for 3 min, followed by 12 cycles of denaturation at 95°C for 30 s, annealing at 60°C for 30 s, elongation at 72°C for 30 s, and with a final elongation step at 72°C for 5 min. Aliquots of indexing-PCR amplicons were separated by 1.5% (wt/vol) agarose gel electrophoresis (100 V, 1 h) in TBE buffer, and gels photographed on a transilluminator (Bio-Rad Laboratories, Vienna, Austria). The intensity of DNA bands was measured using Image Lab 6.1 software (Bio-Rad Laboratories) to generate pooled libraries of amplicons mixed in equimolar concentrations. Libraries were first cleaned by extraction with phenol–chloroform–isoamyl alcohol (24:24:1, vol/vol/vol) and chloroform–isoamyl alcohol (24:1, vol/vol), followed by two steps of spin filtration using Amicon Ultracel 30K centrifugal filters (Millipore UFC503096) and 500 μl of aUPW. Finally, libraries were purified using Agencourt AmPure XP magnetic beads (Beckman Coulter GmbH, Krefeld, Germany) according to the manufacturer’s instructions, and DNA quality and quantities were tested with a 2100 Bioanalyzer (Agilent, Santa Clara, CA, U.S.A.) and adjusted to 10 ng. Library denaturation, addition of internal control DNA (PhiX), and sample loading followed the Illumina protocol. Sequencing was performed on a MiSeq desktop sequencer (Illumina) using a v3 reagent kit (600 cycles). Controls included in MiSeq analyses comprised DNA isolated from 75 μl of thawed mock community suspension (ZymoBIOMICS Microbial Community Standard, Zymo Research, Irvine, CA, U.S.A.) to test the efficacy of DNA isolation, PCR amplification, and sequencing. To exclude contaminations alongside sample preparation, the negative controls of each PCR reaction, whereby PCR-grade water replaced template DNA, and pieces of the agarose gels used to isolate bacterial amplicons were also processed and loaded.

#### Sequence data processing and statistical analysis

Amplicon sequence variants (ASVs) of plastidial and mitochondrial origin were removed, and contaminants were filtered out using the decontam R package (Callahan et al. 2022) set at a prevalence threshold of 0.1.

The amplicon sequencing dataset was split into two subsets analyzed separately. The first subset assessed the effect of the factors “seed compartment,” “time of germination progress,” and “cultivar” on bacterial communities’ shifts, whereas the second sub-set dissected the role of the factor ABA “treatment” compared with (i) germinated seeds 5 days after imbibition started (t-germ) and (ii) imbibed seeds before any had germinated (t-imb) and among the three “seed compartments.” In each subset, samples were analyzed following the same workflow. First, bacterial richness (i.e., number of detected ASVs) and evenness (Simpson’s index) values were calculated as components of α-diversity by averaging the results after multiple rarefactions (999 iterations), using the rtk R package for normalization (Saary et al. 2017). Richness and evenness values were jointly analyzed with recursive partitioning analysis, including all factors considered in both subsets and using the partykit R package (Hothorn et al. 2020). Linear regression analyses were then conducted on richness and evenness values, including all factors of both subsets and considering either the main effects alone or main effects and interactions. The accuracy of the linear regression models was assessed with a machine learning approach employing repeated *k*-fold cross-validation, as implemented in the caret R package (Kuhn 2008). The models with higher squared correlation (*R*^2^) and lower root-mean-squared error were chosen for analysis of variance (ANOVA). *Post hoc* pairwise comparisons between the levels of each factor were subsequently performed on estimated marginal means using the emmeans package (Lenth et al. 2020).

Prior to β-diversity analysis of each subset, data were normalized with the median-of-ratios method implemented in the DESeq2 R package to account for differences in sequencing depth (Love et al. 2021). A supervised analysis of β-diversity patterns was performed with constrained analysis of principal coordinates (CAP) on Bray–Curtis dissimilarities, followed by permutational multivariate analysis of variances (PERMANOVA), using the vegan R package (Oksanen et al. 2019). CAP ordination plots were generated using ggvegan (Simpson 2021) and ggplot2 (Wickham et al. 2021).

To identify indicator taxa, a rank test, as implemented in the RVAideMemoire R package, was initially applied to each factor (Hervé 2020). After correction by false discovery rate (FDR), enriched ASVs were selected according to *P* values < 0.05, and their importance was hence assessed with random forest analyses, which included evaluation of model performances with repeated *k*-fold cross-validation (10-fold, 10 repetitions) and parameter tuning with mtry values between 1 and the square root of the total number of ASVs. The package caret R (Kuhn 2008) assisted model training, and mtry values resulting in highest model accuracy were selected for random forest analyses, while the importance of ASVs was chosen with permutations (999 iterations) run on the rfPermute R package (Archer 2022). ASVs with significant mean decrease accuracy (*P* values < 0.05) were extracted and used to generate barplots (RAM R package; Chen et al. 2018).

To ascertain ASVs of the seed “core microbiome” that were transmitted from t-imb to t-germ across all four cultivars, the core.OTU function of the RAM R package (Chen et al. 2018) was run with prevalence and detection threshold (i.e., relative abundance of all ASVs) of 67% (two out of three biological replicates) and 1%, respectively, as previously reported (Buchholz et al. 2019; Pfeiffer et al. 2017). Intersections among the resulting core groups were visualized with the online tool jvenn (Bardou et al. 2014).

The concentrations of metabolites were imported in R for correlation analyses with ASVs and processed using the mikropml R package (Topçouğlu et al. 2021). A PERMANOVA based on Euclidean distances was applied on transformed metabolite concentrations after centering, scaling, and removal of cases with near-zero variance. The importance of both metabolite and bacterial amplicon sequencing data was evaluated through a random forest analysis. A Spearman’s correlation analysis was then performed between criteria-fulfilling metabolites and selected ASVs with the psych R package (Revelle 2022). Following FDR correction of *P* values, correlograms were generated with the corrplot R package (Wei et al. 2021).

### Targeted metabolite profiling and bacterial growth tests

#### Metabolite extraction, derivatization, and GC-MS analyse

Metabolites were extracted from 10.27 ± 0.15 mg of ground embryonic compartments of ‘Abelina’ as detailed in Gerna et al. (2018) and Gerna et al. (2021) and after minor adaptations of the method by Fiehn (2016). Samples, including blanks, were injected randomly, alongside standards prepared from analytical grade amino acids, organic acids, saccharides, and sugar alcohols (Sigma Aldrich, St. Louis, MO, U.S.A.) covering the range of detected concentrations. Metabolite derivatization required two steps with methoxamine hydrochloride in pyridine and *N*-methyl-*N*-trimethylsilyl-trifluoroacetamide prior to split-10 injection of a 1-μl sample into a Trace 1300 gas chromatogram (Thermo Scientific, Waltman, MA, U.S.A.) for separation through a 30-m Rxi-5SilMS column (0.25 μm cross bond) equipped with a 13623-127 10-m Integra-Guard precolumn (Restek, Bellefonte, PA, U.S.A.). The glass liner (23467, Restek) was regularly replaced after injection of a maximum of 25 samples. Metabolites were detected via a coupled TSQ800 triple quadrupole mass spectrometer (Thermo Scientific) operated with electron impact ionization (70 eV) and ion source temperature set at 330°C, with mass spectra acquired in full scan mode from *m/z* 50 to 600 at 5 spectra s^−1^. Raw data files were analyzed with AMDIS software (v2.73 https://chemdata.nist.gov/dokuwiki/doku.php?id=chemdata:amdis; Stein 1999), operated at a medium resolution, sensitivity, and shape requirement, with a minimal signal-to-noise ratio set to 80. Metabolites were identified through direct comparison of retention indices and ion fragmentation with external standards. Peak areas of selected trace ions (Supplementary Table S1) were integrated using Xcalibur v2.2 (Thermo Scientific) with the Genesis algorithm and quantified with standard calibration curves after normalizing to a ^13^C_6_-sorbitol internal standard (Campro Scientific, Berlin, Germany) and sample DW. Molar concentrations of metabolites were calculated using the water content of each seed and seedling compartment. For the whole seedling axis, weighted average concentrations were calculated from those of the separately measured radicle and nonemerged plumule and considering their difference in water volume (0.74: 0.26, radicle: plumule).

#### Bacterial growth tests on minimal tailored media

The molar concentrations of the most abundant three or four amino acids, saccharides, organic acids, and sugar alcohols measured in both cotyledons and seedling axes of t-germ seeds (Supplementary Table S2) guided the design of minimal M9 solid-based media (1% w/v agar) for culturing selected bacterial strains, following a previously developed approach (Gerna et al. 2022). The strains *Rhodococcus fascians* 20669 (99.5% homology with ASV 88) and *Pantoea agglomerans* 1619 (98.0% homology with ASV 3) were obtained from the Leibniz Institute DSMZ—German Collection of Microorganisms and Cell Cultures GmbH (Braunschweig, Germany). Due to cell aggregation in liquid media hindering absorbance measurements of culture density, a solid media approach was used. Single colonies (*n* = 4 for each strain) were loop inoculated in 10% (wt/vol) tryptic soy broth (TSB, Corning, Manassas, VA, U.S.A.) and grown at 28°C and 180 rpm for 48 to 72 h. After TSB was removed by centrifugation (7,500 × *g*, 10 min), pellets were washed twice in M9 minimal salts medium (Formedium, Hunstanton, U.K.) and adjusted to 10^6^ CFU ml^−1^. Suspensions were 10-fold serially diluted in M9 minimal salts medium and plated on M9 medium only, M9-based media supplemented with metabolites of each biochemical class at estimated molar concentrations, and 10% (wt/vol) tryptic soy agar (TSA), a nutrient-rich medium used as control. Changes in the number of colony-forming units were recorded following incubation at 23°C until no changes occurred within two consecutive days.

#### Statistical analysis

Data distribution was initially inspected for normality and homoskedasticity through quantile–quantile plots, Shapiro–Wilk, and Levene’s tests, to reveal differences in the metabolite concentrations measured in both embryonic compartments at t-dry, t-imb, and t-germ. In the absence of normally distributed residues, concentrations were assessed for significance (α= 0.05) by running multiple nonparametric Mann–Whitney *U* tests with ties correction for each combination of metabolite, embryonic compartment, and time of germination progress, using the software package SPSS Statistics 25 (IBM, New York). The same workflow was implemented to assess the significance of the colony-forming units number by using nonparametric Kruskal–Wallis rank variance analysis followed by the Bonferroni correction.

## Results

### Seed compartment and germination progress greatly affected the α-diversity of bacterial communities

An average of 55,916 high-quality merged nonchimeric reads per sample with a median length of 374 bp was retrieved after sequencing. In dry seeds, the top 10 most abundant bacterial families were mainly attributed to the Actinobacteria and α-Proteobacteria, and only partially to the γ-Proteobacteria and Bacteroidia (Supplementary Fig. S2; Supplementary Table S3). About 99% of the ASVs assigned to such families were detected on the seed coats of dry seeds, with the notable exception of the Morganellaceae, which, with the genus *Buchnera*, were predominantly hosted in the embryonic compartments (Supplementary Table S3). The potential bacterial inoculum for the adult holobiont found in dry seeds started to uncover dynamic shifts of ASVs in relation to three factors: (i) “seed compartment,” consisting of embryos (embryonic/seedling axis, cotyledons) and seed coats; (ii) “time of germination progress,” referring to dry seeds (t-dry), seeds that had imbibed before any had germinated (t-imb), and germinated seeds 5 days after imbibition started (t-germ) with >1 cm radicle and green nonemerged epicotyl (plumule); (iii) “cultivar,” including Abelina, Amadea, Amadine (from organic farming), and Cordoba. Machine learning analyses enabled selection of the best linear regression model (i.e., ANOVAs with or without interactions; Supplementary Table S4) to assess α-diversity, which overall supported the outcomes of recursive partitioning analysis (Supplementary Fig. S3). In both cases, the main factor affecting either richness or evenness of bacterial microbiomes was “seed compartment,” followed by “time of germination progress” and their interaction (Supplementary Fig. S3; Supplementary Table S4).

By t-imb, bacterial richness increased progressively in both embryonic compartments, while dropping about 1.5-fold in seed coats (Supplementary Fig. S3). Changes in communities’ evenness reflected those in richness in all seed compartments. The contribution of “cultivar” to α-diversity was marginal overall and did not significantly affect the α-diversity of bacterial microbiomes as main effect (Supplementary Table S4).

In summary, embryonic compartments and seed coats displayed contrasting changes in α-diversity as germination advanced regardless of cultivar.

### β-diversity of bacterial microbiomes homogenized across seed compartments during the progress of germination

**Fig. 2:**
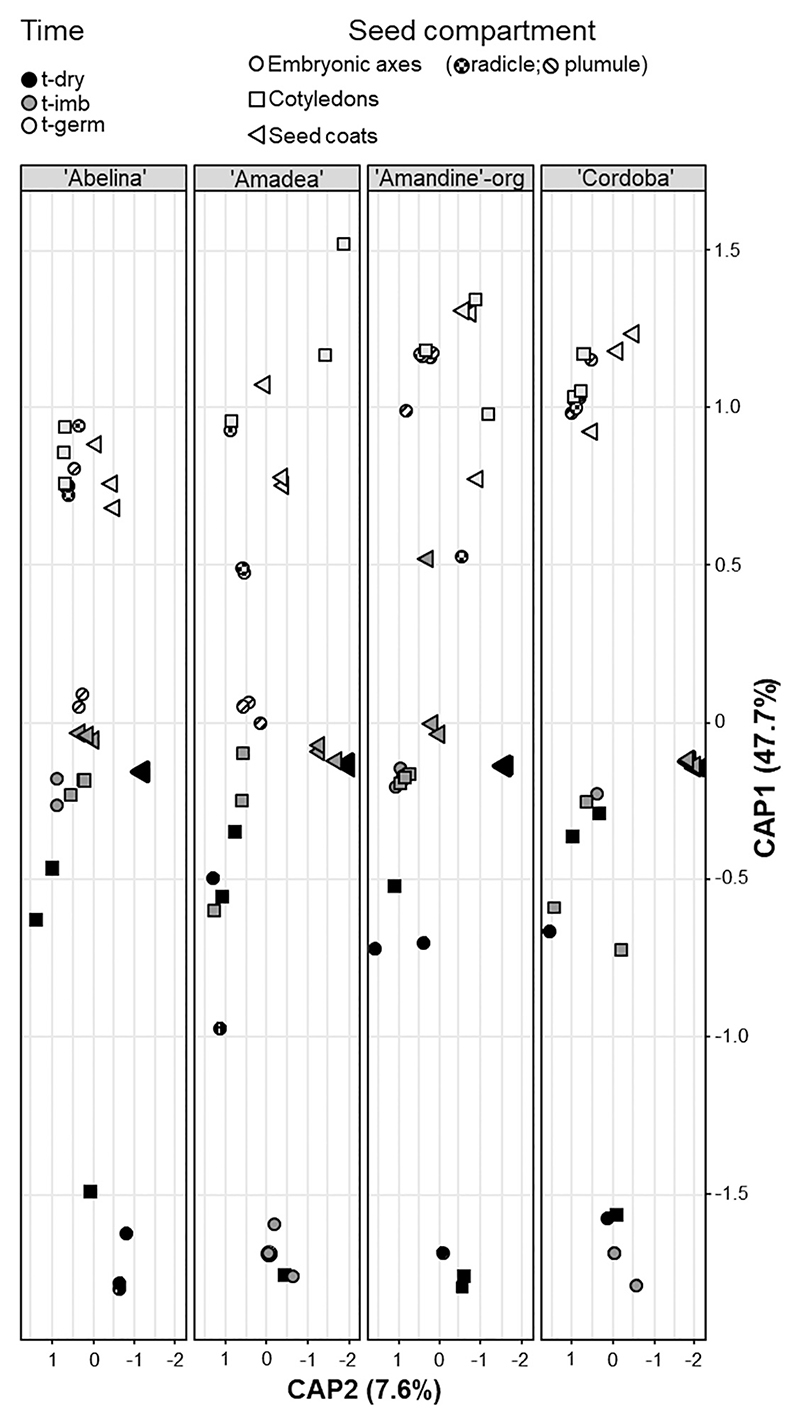
Constrained analysis of principal coordinates (CAP) performed on Bray–Curtis dissimilarities on normalized data, considering “time of germination progress” and “seed compartment” (see key, top) as constraining factors for each of the four soybean cultivars indicated in gray boxes. t-dry, dry seeds; t-imb, imbibed seeds just before any had germinated; t-germ, germinated seeds 5 days after imbibition started. Each symbol represents a unique replicate (*n* = 3 of 30 seeds each). The proportions of variance explained by constrained eigenvalues are reported along the figure axes.

**Fig. 3:**
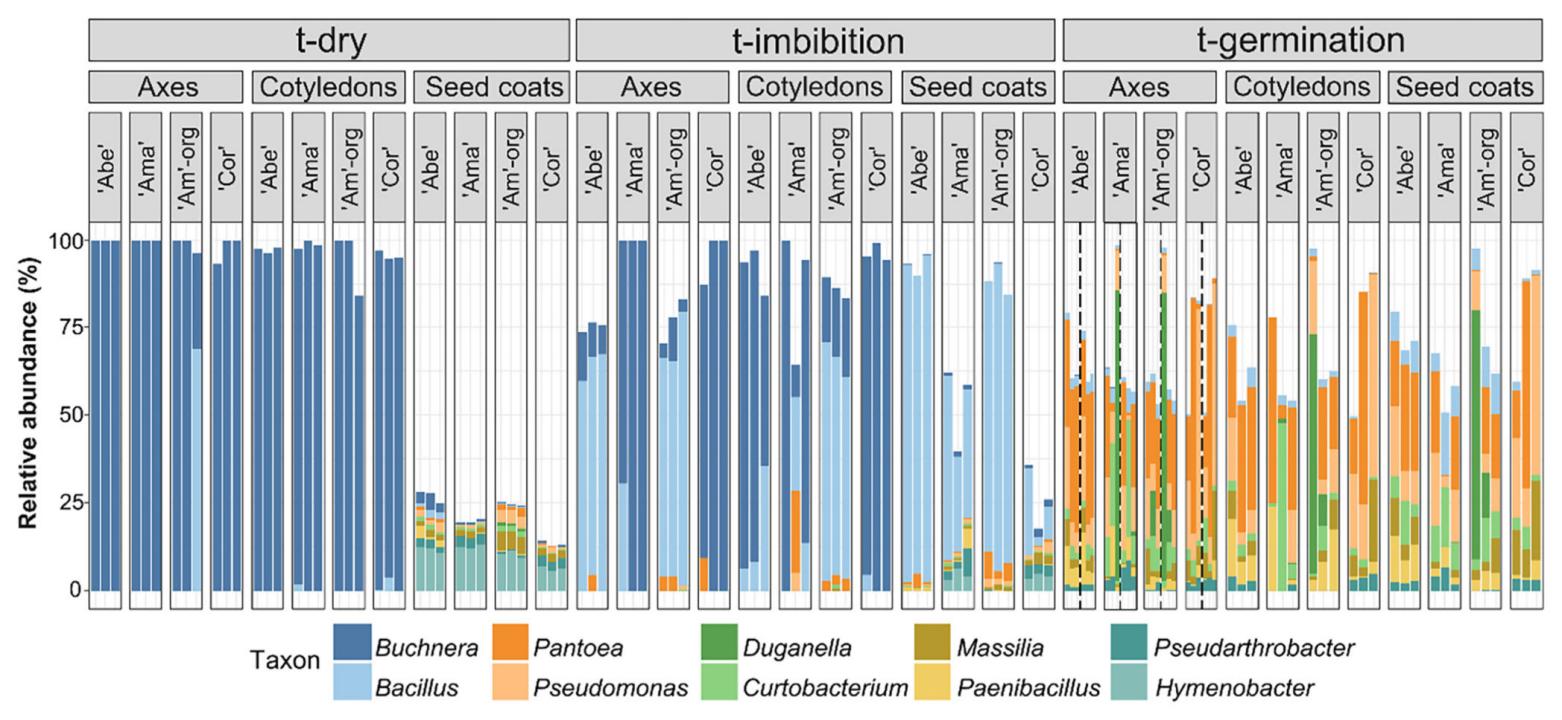
Relative abundance of the top 10 bacterial indicator genera influencing the composition of microbiomes during the progress of soybean seed germination. Vertical bars represent replicates (*n* = 3 of 30 seeds) ordered by the factors (i) “time of germination progress”: dry, dry seeds; t-imbibition, imbibed seeds just before any had germinated; t-germination, germinated seeds 5 days after imbibition started. (ii) “Seed compartment”: cotyledons, seed coats, and embryonic/seedling axes (axes) that were subdivided into radicle and nonemerged plumule (left and right of dashed lines, respectively) at t-germination. (iii) “Cultivar”: Abelina (Abe), Amadea (Ama), Amadine organic (Am-org), and Cordoba (Cor). Distinctive colors denote genera (bottom key).

The analysis of β-diversity showed that “time of germination progress” (pseudo-*F*: 34.473, *P* value < 0.001) and “seed compartment” (pseudo-*F*: 21.547, *P* value < 0.001) were the main factors differentiating the structure of bacterial microbiomes, with “cultivar” displaying minor but still significant influences (pseudo-*F*: 5.001, *P* value < 0.001; Supplementary Table S5). Notably, the compositional dissimilarity of bacterial microbiomes of seed compartments tended to attenuate while germination advanced (Supplementary Fig. S4), whereas “time of germination progress” became increasingly important in structuring the microbiome of each seed compartment (Supplementary Table S6). Overall, bacterial communities at t-dry revealed conspicuous dissimilarity to t-imb and t-germ (Fig. 2). The relative abundance of indicator taxa, which were selected based on the most abundant ASVs that accounted for the structure of bacterial communities, confirmed this pattern (Fig. 3). At t-dry, embryos were predominantly characterized by the genus *Buchnera* (on average 95%), whereas seed coats harbored a higher diversity of genera at low relative abundance, including *Hymenobacter* (10%), *Massilia* (3%), *Pseudarthrobacter* (2%), and *Pseudomonas* (1%) (Fig. 3; Supplementary Table S7). Furthermore, ASVs at t-imb of both seed embryonic compartments started to cluster with those of seed coats, as visualized through a CAP plot (Fig. 2). Such homogenization was also reflected by changes of the indicator taxa, which overlapped across all seed compartments at t-germ (Fig. 3). Notably, genera identified on seed coats at t-dry (e.g., *Pantoea, Pseudomonas, Bacillus*, and *Curtobacterium*) were enriched in all seed compartments at t-germ. In contrast, the indicator taxon *Buchnera*, which dominated the embryonic communities at t-dry, was hardly found at t-germ (Fig. 3; Supplementary Table S7). When the core microbiome comprising only ASVs found in all four cultivars is considered, no unique ASV at t-germ originated exclusively from embryonic compartments at t-imb, while all ASVs at t-germ were the same across all seed compartments, assigned to the Bacillales (~27%), Micrococcales (~16%), and Enterobacterales (~11%), followed by Burkolderiales, Paenibacillales, and Pseudomonadales (each at ~7%; Supplementary Fig. S5; Supplementary Table S8).

### Influence of exogenous ABA on seed germination, metabolites, and bacterial communities

The phytohormone ABA is an endogenous inhibitor of seed germination. When provided exogenously to seeds of ‘Abelina’, the most cold- and ABA-sensitive cultivar in this study (Clara 2021), 100 μM ABA slowed average germination time, but total germination % was hardly reduced (Supplementary Fig. S6), and 500 μM reduced total germination to ~60% after 15 days, whereas 800 μM prevented germination until 14 days, and thereafter total germination did not exceed ~ 20% (Supplementary Fig. S6). However, imbibition was not at all inhibited by 800 μM ABA (Supplementary Fig. S6). Seeds imbibed with 800 μM ABA for 5 days, the time point when they otherwise would have already germinated (i.e., t-germ), are hereafter referred to as t-germ-ABA.

**Fig. 4:**
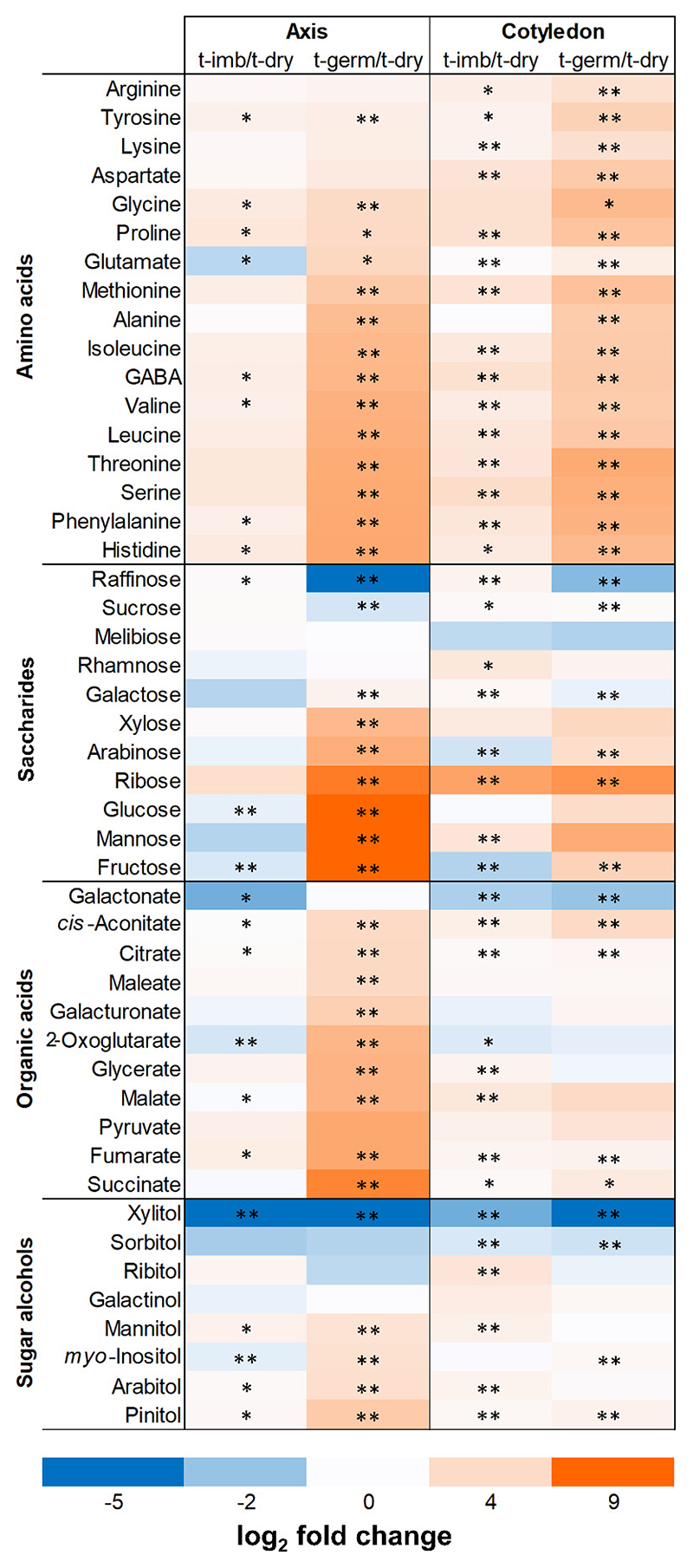
Metabolite changes during seed germination of the soybean cultivar Abelina. Seed metabolites are grouped into classes of amino acids (with *γ*-aminobutyric acid, GABA), saccharides, organic acids, and sugar alcohols. Seeds were analyzed before imbibition (t-dry), after 50 h imbibition before any had germinated (t-imb), and germinated 5 days after imbibition started (t-germ). Fold changes in the concentrations of metabolites between t-imb and t-dry, and t-germ and t-dry, respectively, are shown for cotyledon and embryonic/seedling axis (axis) on a log_2_ basis with colored scale (bottom). Asterisks denote significant differences at *P* value < 0.05 (single) and < 0.01 (double) for each metabolite relative to concentrations at t-dry.

**Fig. 5:**
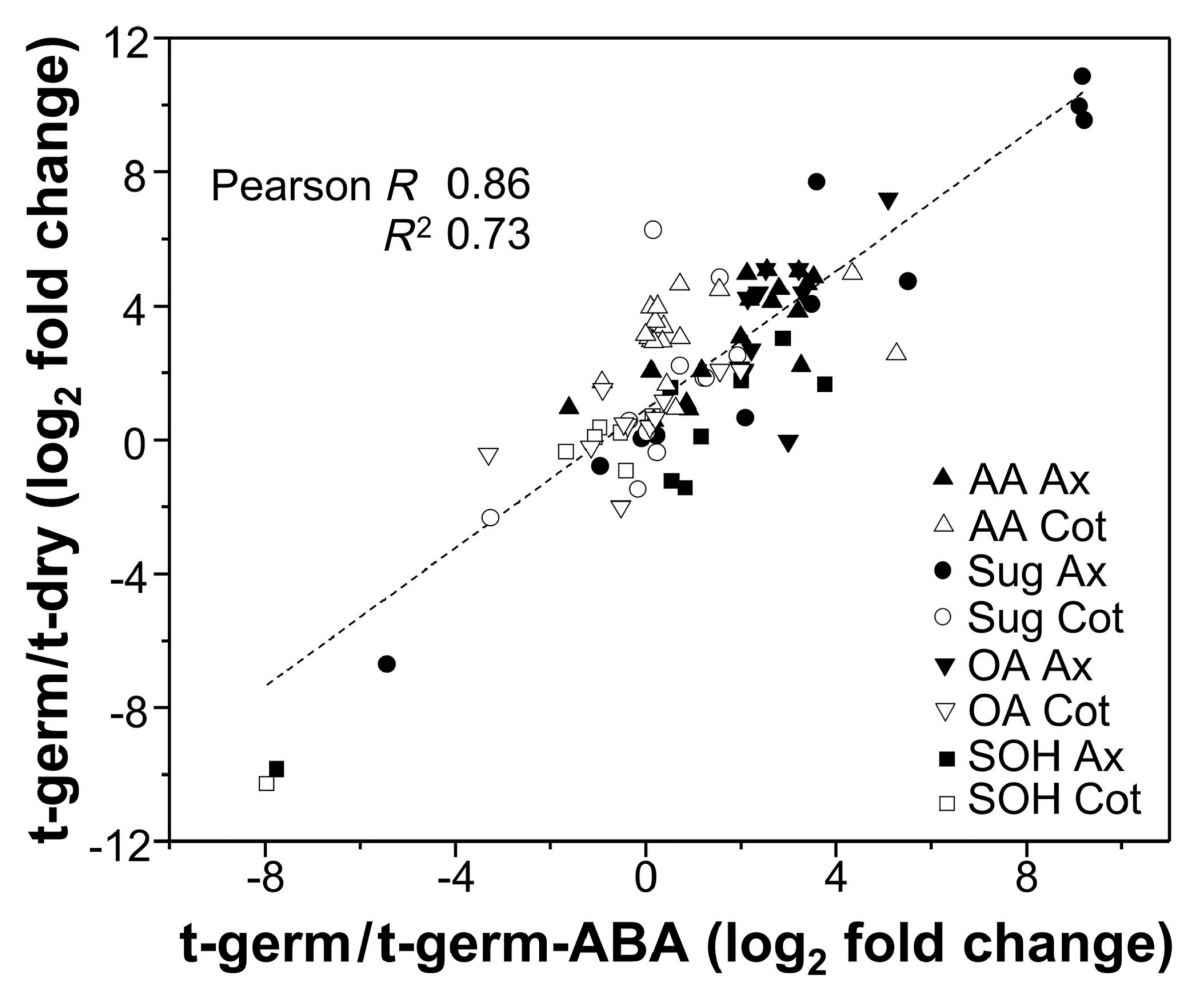
Correlation between changes in metabolite concentrations 5 days after imbibition started (t-germ) relative to either seeds imbibed with 800 μM abscisic acid (t-germ-ABA) or dry seeds (t-dry). Symbols denote biochemical classes of metabolites in embryonic/seedling axes (closed symbols; Ax) and cotyledons (open symbols; Cot). The linear fit considers all data (*n* = 5 replicates of 30 axes or cotyledons for each time point of germination progress). Triangles, amino acids (AA); circles, saccharides (Sug); inverted triangles, organic acids (OA); squares, sugar alcohols (SOH).

To reveal metabolite changes with potential relevance to bacterial growth and microbiomes’ shifts in the seed endosphere during germination, GC-MS-based targeted profiling of 47 primary metabolites, including amino acids, saccharides, organic acids, and sugar alcohols, was conducted. Overall, most metabolites accumulated in at least one seed compartment at t-imb, especially in the cotyledons (Fig. 4). At t-germ, except for raffinose, galactonate, xylitol, and sorbitol, which were depleted, almost all metabolites had increased in the cotyledons and/or seedling axis (i.e., protruded radicle and nonemerged plumule; Fig. 4). Sucrose, glutamate, citrate, and pinitol were the most concentrated saccharide, amino acid, organic acid, and sugar alcohol, respectively, in the cotyledons, as well as highly abundant metabolites in the seedling axis (Supplementary Fig. S7). The metabolite changes between t-dry and t-germ were greatly impeded by imbibition with 800 μM ABA (Supplementary Fig. S7). Therefore, the fold difference of each metabolite concentration at t-germ-ABA relative to that at t-germ was very similar to the fold difference of the same metabolite at t-dry relative to that at t-germ, leading to a strong positive linear correlation with the Pearson R coefficient of 0.86 when all 47 metabolites were considered (Fig. 5). In summary, metabolism that had occurred between t-dry and t-germ was severely restricted by exogenous ABA.

**Fig. 6:**
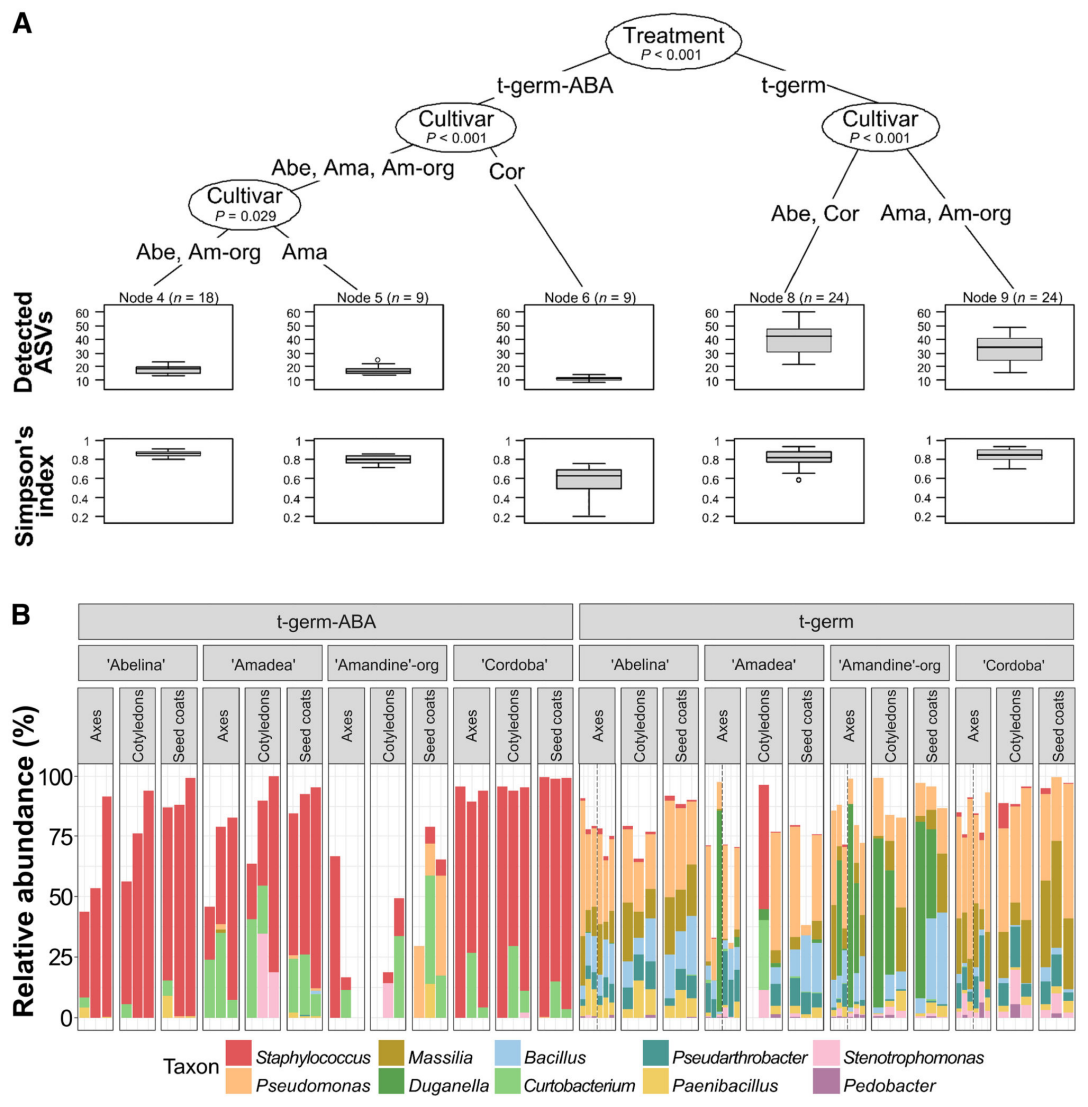
Effect of exogenous abscisic acid (ABA) on the richness and diversity of bacterial communities of soybean seeds 5 days after imbibition started. **A**, Decision tree of bacterial α-diversity based on recursive partitioning analysis. The factors are (i) “treatment”: ABA-treated seeds (t-germ-ABA) and untreated control (t-germ); (ii) “cultivar”: Abelina (Abe), Amadea (Ama), Amadine organic (Am-org), and Cordoba (Cor). Richness, as represented by number of detected amplicon sequence variants (ASVs), and diversity, calculated as Simpson’s index, are shown as boxplots with outliers denoted by open circles. **B**, Relative abundance of the top 10 bacterial indicator genera (bottom key) influencing the microbiomes’ composition in embryonic and seedling axes (axes), cotyledons, and seed coats of each cultivar, with t-germ seedling axes subdivided into radicle and nonemerged plumule (left and right of dashed lines, respectively). Each bar represents a unique replicate (*n* = 3 of 30 seeds each).

In relation to α-diversity, at t-germ “treatment” with ABA was the major factor affecting bacterial richness (Fig. 6A), which was on the average across cultivars >2-fold lower in t-germ-ABA seeds than in t-germ seeds (*F*-value: 136.586, *P* value < 0.001; Supplementary Table S9). Furthermore, in t-germ-ABA seeds, communities became less evenly distributed (*F*-value: 8.867, *P* value < 0.005; Supplementary Table S9), as viewed through a lower Simpson’s index. Concerning β-diversity, ABA “treatment” was also the major driver separating the structure of bacterial communities (pseudo-*F*: 14.525, *P* value < 0.001; Supplementary Table S10), which homogenized across seed compartments, as observed previously without ABA treatment, and also indicated by “compartment” making a weaker contribution to clustering in a CAP plot (Supplementary Fig. S8; Supplementary Table S10). The deeply reduced bacterial richness of t-germ-ABA in contrast to t-germ seeds (Fig. 6A) also resulted in a smaller number of indicator taxa that were assigned to the Staphylococcaceae (ASVs 9, 164, and 406), Bacillaceae (ASV 29), and Microbacteriaceae (ASV 53; Fig. 6B; Supplementary Table S11). The taxonomic homogenization among seed compartments appeared more evident when t-germ-ABA seeds were compared with t-imb seeds (fully imbibed, but not germinated in both cases) due to lower richness than that at t-germ (Supplementary Fig. S9). *Staphylococcus* and *Curtobacterium* mostly distinguished the communities of t-germ-ABA seeds from t-germ seeds (Fig. 5B; Supplementary Fig. S9; Supplementary Table S12), which extended to *Pantoea* and *Frigoribacterium* when compared with t-imb seeds (Supplementary Fig. S9; Supplementary Table S12).

In summary, seed imbibition with ABA limited the increase of bacterial richness, but not taxonomic homogenization of bacterial microbiomes between seed compartments.

### Metabolites that accumulated in germinating seeds stimulated rapid growth of copiotrophs

**Fig. 7:**
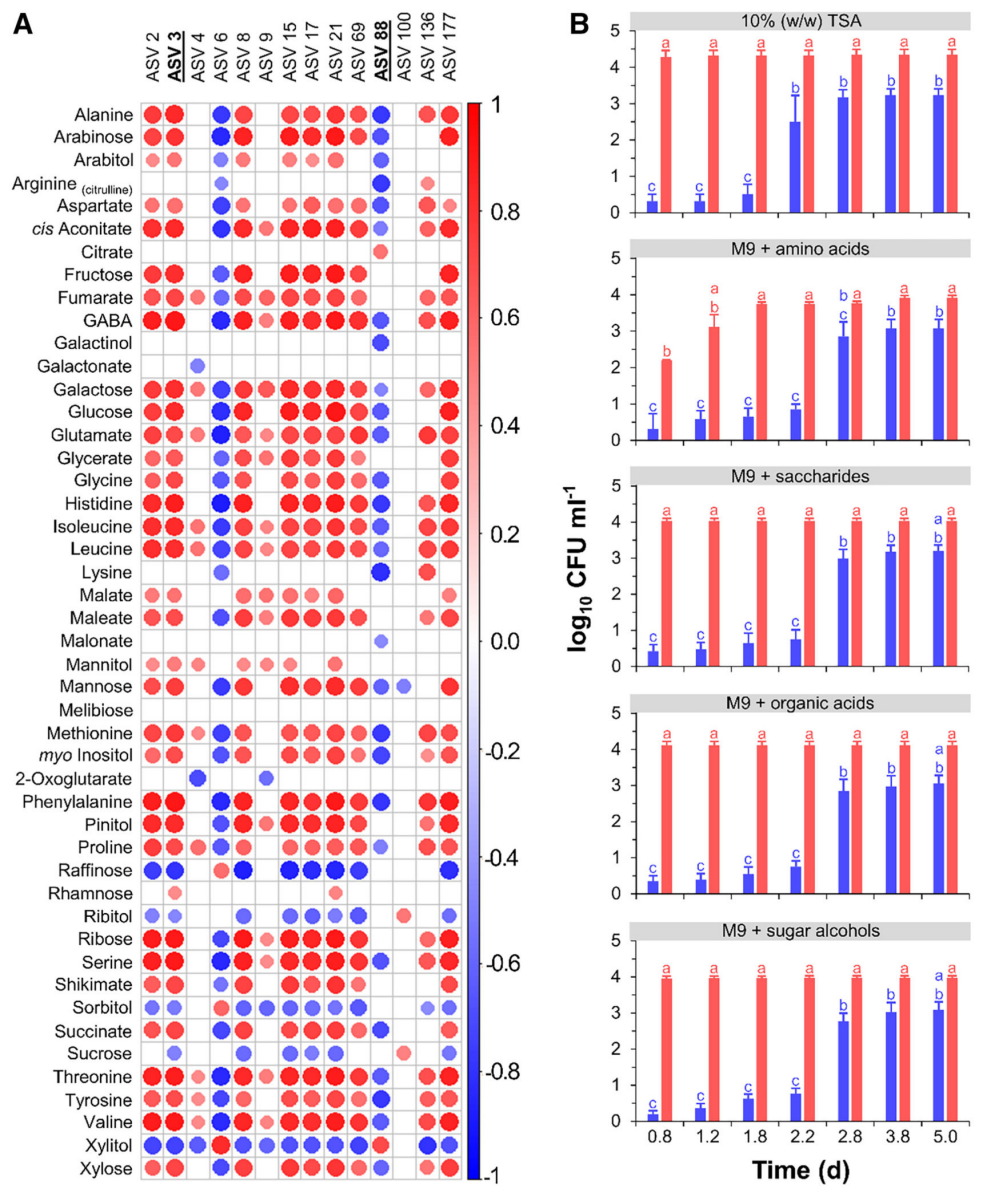
Correlations of seed metabolite changes with shifts in bacterial taxa during the progress of soybean seed germination, and influence of metabolites on bacterial growth. **A**, Correlogram summarizing correlations between individual metabolites and amplicon sequence variants (ASVs) selected according to their abundance (rank test) and importance (random forest), as given in Supplementary Table S7. Significant correlations are depicted by circles, whose diameter is proportional to Spearman’s *r* coefficients (*P* value < 0.05), and colors refer to the correlation direction (key on the right). Empty cells denote lack of significant correlation. Taxonomic assignment: ASV 2, Erwiniaceae; ASV 3, *Pantoea agglomerans*; ASV 4, *Bacillus*; ASV 6: *Buchnera aphidicola*; ASV 8, *Curtobacterium*; ASV 9, *Staphylococcus*; ASV 15, *Bacillaceae*; ASV 17, *Paenibacillus*; ASV 21, *Pseudomonas*; ASV 69, Enterobacterales; ASV 88, *Rhodococcus fascians*; ASV 100, *Bacillus halmapalus*; ASV 136, Bacillales; ASV 177, *Paenibacillus*. **B**, Changes over time in the number of colony-forming units (CFU) of selected strains on tryptic soy agar (TSA) and M9 minimal saltsbased media supplemented with the most abundant metabolites targeted in each biochemical class, as indicated in gray headings, at concentrations detected in the cotyledons of germinated seeds 5 days after imbibition started. Data are means ± SE (*n* = 4) for *Rhodococcus fascians* (ASV 88, blue) and *Pantoea agglomerans* (ASV 3, red), and different letters denote significant differences (nonparametric Kruskal–Wallis rank tests followed by the Bonferroni correction; *P* value < 0.05).

Rank tests on the relative abundance of ASVs, supported by random forest algorithms across “seed compartment,” “time of germination progress,” and “cultivar,” pinpointed 14 ASVs whose importance and abundance correlated significantly (*P* value < 0.05) with changes in seed metabolite concentrations during the progress of germination (Fig. 7A; Supplementary Table S13). Most of these ASVs were attributed to common seed-associated families (e.g., Enterobacteriaceae) and correlated positively with the metabolites that accumulated in the seed endosphere. Negative correlations were established for two ASVs only, which were assigned to *Buchnera* (ASV 6) and *Rhodococcus* (ASV 88) (Fig. 7A; Supplementary Table S13).

Based on their opposite correlations, we selected ASV 3 (*Pantoea*) and ASV 88 (*Rhodococcus*) for monitoring of growth on minimal media tailored to the metabolite composition of soybean cotyledons and axes of t-germ seeds (Fig. 7A; Supplementary Fig. S7). The two strains *P. agglomerans* 1619 and *R. fascians* 20669, showing >98% sequence identity with ASV 3 and ASV 88, respectively, were chosen as representative taxa with abundance positively and negatively correlating with metabolite changes. When M9 medium was supplemented with any of the most abundant metabolites at concentrations measured in t-germ cotyledons, *P. agglomerans* grew very fast, and within about 1 day reached a plateau number of colony-forming units, regardless of whether supplied with amino acids, saccharides, organic acids, or sugar alcohols, hence revealing metabolic flexibility (Fig. 7B). In contrast, the number of *R. fascians* colony-forming units progressed more slowly and stabilized after 3 days (Fig. 7B). This trend was similar irrespective of whether metabolite concentrations (Supplementary Table S2) mimicked those detected in t-germ cotyledons or t-germ seedling axes, excluding a medium containing low concentrations of three organic acids (as found in t-germ axes) as sole carbon sources, which was not able to sustain rapid growth of either strain (Supplementary Fig. S10).

## Discussion

Mature dry seeds harbor a lower endophytic bacterial richness and taxonomic diversity than other plant organs (Eyre et al. 2019; Hardoim et al. 2015; Truyens et al. 2015), which has been related to scarce microbial niches and limited water and nutrient availability relative to later plant developmental stages (Dutta et al. 2022; Liu et al. 2013; Okunishi et al. 2005). Thus, imbibition and resumption of seed primary metabolism during germination appear likely to be critical for initial assembly of the plant holobiont that arises from a seed (Nelson 2018; Shade et al. 2017; Simonin et al. 2022). Here, we have revealed a role for seed imbibition in homogenizing bacterial microbiomes across seed compartments, while highlighting the importance of seed germinative metabolism on modelling bacterial richness and composition of the holobiont.

### Spatial separation of bacterial communities attenuated during the progress of seed germination

Different developmental stages, including seed maturation drying, germination, and seedling emergence, can deeply shape the diversity and structure of seed bacterial communities (Barret et al. 2015; Chesneau et al. 2020; Torres-Cortés et al. 2018). Likewise here, the transition from dry (t-dry) to imbibed (t-imb) soybean seeds distinctly affected epiphytic and endophytic bacterial microbiomes. To persist before imbibition, endophytic bacteria must be able to tolerate the high cellular viscosity, inert storage reserves, and low water availability typical of dry desiccation tolerant seeds (Ballesteros et al. 2020), as well as achieving vertical inheritance from plant to seed (Abdelfattah et al. 2023; Hardoim et al. 2015; Shahzad et al. 2018). In contrast, the epiphytic seed coat communities can originate from horizontal transmission, simply by erratically landing on the seed or fruit (Nelson 2018). In t-dry seeds of all cultivars, seed coats displayed higher richness and evenness than the endophytic communities of the embryonic compartments (Supplementary Fig. S3). Notably, ASVs assigned to *Buchnera* were prominent in both embryonic compartments of dry seeds of all cultivars (Fig. 3; Supplementary Table S8), although this genus has been rarely reported to inhabit the seed endosphere (Majumdar et al. 2021). Considering that aphids are an emerging soybean pest that host *Buchnera* as an obligate intracellular symbiont (Douglas 1998; Gunadi et al. 2017; Hill et al. 2010), its substantial presence as a seed endophyte argues for insectmediated vertical transmission through the phloem sap in cultivars produced under conventional and organic farming practices.

The physicochemical properties of the seed endosphere change progressively with imbibition, as initiated by water movement through cracks on the dorsal surfaces of soybean seeds (Ma et al. 2004). At t-imb, the enrichment of embryonic communities with similar composition across cultivars (Fig. 3; Supplementary Fig. S3) coexists with quiescent forms of bacteria (e.g., endospores, cysts) that persist in the endospheres of dry seeds and reactivate proliferation upon rehydration (Cope-Selby et al. 2017; Gerna et al. 2022; Mano et al. 2006). Current knowledge on the desiccation tolerance strategies of seed-associated bacteria remains limited, but *Bacillus*, at least, is well known for sporulation (Beskrovnaya et al. 2021), which may partly explain its rapid spread in the communities of t-imb seeds (Fig. 3).

Plant genotype and parental environment, including agricultural practice, can influence seed bacterial endophytic (Adam et al. 2018; Chen et al. 2020; Escobar Rodríguez et al. 2020; Eyre et al. 2019; Johnston-Monje and Raizada 2011; Klaedtke et al. 2016; Rezki et al. 2018; Rochefort et al. 2019) and epiphytic (Links et al. 2014) communities. “Seed compartment” was by far the most influential factor that differentiated the bacterial microbiomes of dry seeds (Fig. 2; Supplementary Fig. S3), overruling the contribution of “cultivar” and any associated differences related to the distinct location of seed production, including organic and nonorganic agricultural practices. However, the role of “seed compartment” in distinguishing microbiomes was attenuated with germination, as revealed by the overlapping microbiomes of seedling axes, cotyledons, and seed coats in all cultivars at t-germ (Figs. 2 and 3). The core microbiome offered no evidence that ASVs of t-germ seeds preferentially originated from a certain seed compartment of t-imb seeds (Supplementary Fig. S5), but instead several indicator genera with low relative abundance on dry seed coats (i.e., epiphytes) became dominant in all seed compartments at t-germ, and consistently for all cultivars (Fig. 3). Thus, bidirectional endophytic–epiphytic lifestyle switches may also account for such taxonomic homogenization of the bacterial microbiomes, as previously suggested (Gerna et al. 2020; Hardoim et al. 2012; Nelson 2018). Particularly, bacterial responses to the rapidly changing seed microhabitats during germination included epiphytes colonizing the endosphere, and endophytes redistributing up to the seed surface.

Bacterial taxa also homogenized among seed compartments in imbibing but nongerminating ABA-treated seeds (Fig. 6A). *Staphylococcus* and *Curtobacterium* dominated in t-germ-ABA seeds (Fig. 6B; Supplementary Fig. S5), and isolates of these genera cultured from the same seed lots studied here have the capacity to influence germination under salt stress (Gerna et al. 2022), which stimulates ABA biosynthesis (Wang et al. 2015). Such genera are frequently reported in association with seeds (Alibrandi et al. 2018; Bziuk et al. 2021; Dunleavy 1989; Lopez-Velasco et al. 2013; Torres-Cortés et al. 2018), and their prominent abundance in t-germ-ABA seeds also suggests high competitiveness under restricted nutrient availability imposed by exogenous ABA.

To summarize, homogenization of bacterial taxa across seed compartments and a weakening of niche boundaries between epiphytes and endophytes could still occur without seed germinative metabolism, revealing imbibition alone as the earliest step in assembling the plant holobiont.

### Seed metabolism fuels the richness of seed microbiomes: Relevance of bacterial life strategies

During seed germination, metabolite changes occurred in the embryo (Fig. 4; Supplementary Fig. S7), showing resumption of plant energetic metabolism and seed reserve mobilization (Bau et al. 1997; Gu et al. 2017). Most primary metabolites rose by several fold at t-germ (Fig. 4), apart from those typically implicated in desiccation tolerance (e.g., raffinose, xylitol), which declined. Exogenous ABA minimized seed metabolite changes, without interfering with soybean seed water uptake (Fig. 5; Supplementary Fig. S6), thus enabling to distinguish the influence of imbibition from that of seed metabolism on shifts in bacterial microbiomes. Drops in the relative abundance of Bacillaceae and Staphylococcaceae, accompanied by proliferation of Pseudomonadaceae and Enterobacteriaceae during and post-germination, have been reported (Barret et al. 2015; Lopez-Velasco et al. 2013; Torres-Cortés et al. 2018). Our results agreed with such shifts (Fig. 3), whereby generalist genera such as *Pantoea* and *Pseudomonas*, followed by *Paenibacillus* and *Massilia*, outcompeted *Buchnera, Staphylococcus*, and partially *Bacillus*. Exogenous ABA largely impeded these dynamics, and microbiome richness (a proxy of bacterial proliferation) was severely curtailed (Fig. 6A; Supplementary Table S9), supporting that seed germinative metabolism is critical for the development of the seedling holobiont. Since bacteria lack the ABA signaling of plants, we do not expect that bacterial growth would have been restricted by exogenous ABA. Nonetheless, some bacteria can use ABA as a sole carbon source and modulate ABA concentrations in planta (Belimov et al. 2014). Therefore, a direct influence of exogenous ABA on seed bacterial microbiomes’ assemblage cannot be excluded in our study.

Taxonomic shifts in seed microbiomes have been associated with “copiotrophy” and “oligotrophy,” two opposing bacterial life strategies resulting in rapid and minimal growth rates, respectively, in response to local and transitory increases in carbon sources (Barret et al. 2015; Ho et al. 2017; Koch 2001; Torres-Cortés et al. 2018). At t-germ, most primary metabolites had greatly accumulated (Fig. 4), indicating mobilization of seed nutrient reserves from cotyledons to sustain heterotrophic seedling growth. As *Pantoea, Pseudomonas*, and *Massilia* are described as copiotrophs (Barret et al. 2015; Ofek et al. 2011; Torres-Cortés et al. 2018), our data support the conclusion that the functional trait of copiotrophy contributed to increasing richness of endophytic communities in germinated seeds. Selected strains that matched influential ASVs either positively or negatively correlating with metabolite changes associated with germination showed distinct growth rates on minimal media, mimicking the trophic conditions of t-germ seeds (Fig. 7; Supplementary Fig. S10). Therefore, following seed metabolic resumption, slow-growing oligotrophic taxa (e.g., *R. fascians*) appear more prone to be outcompeted by faster-growing copiotrophic taxa (e.g., *P. agglomerans*).

In conclusion, we propose the following route for the establishment of bacterial microbiomes during seed germination. Initially, imbibition enables distribution of seed-coat-derived epiphytes, including copiotrophs, into hydrated embryos. Here, hydration also reactivates metabolite changes participating to seed reserve mobilization and assists the enrichment of fast-growing copiotrophs that eventually predominate as endophytes in germinated seeds. As homogenization of bacterial taxa still occurred in the presence of exogenous ABA, we infer that seed water uptake due to imbibition alone, regardless of metabolite concentrations, is sufficient to redistribute epiphytic and endophytic bacteria across embryonic and nonembryonic compartments. A definition of endophytic lifestyle merely based on the colonization of a certain anatomical niche hence appears as particularly transitory in imbibing seeds, whereas the functional trait of copiotrophy aids bacterial endophytic colonization in the initial steps of the postdispersal assemblage of the plant holobiont.

## Supplementary Material

Supplementary Figures

Supplementary Tables

## Data Availability

Raw 16S rRNA gene sequencing data are available at https://www.ncbi.nlm.nih.gov/sra/PRJNA915932. Negative and positive controls for the 16S rRNA sequencing can be found at https://zenodo.org/record/8211810 and https://zenodo.org/record/8211833, respectively.
