# Supplementary material for: Seed Imbibition and Metabolism Contribute Differentially to Initial Assembly of the Soybean Holobiont
Source: Phytobiomes J. Author manuscript; Available in PMC 2024 May 30. (PMC7616048; doi:10.1094/PBIOMES-03-23-0019-MF)
Supplement: Supplementary Figures [file EMS196334-supplement-Supplementary_Figures.pdf]

SUPPLEMENTARY FIGURES

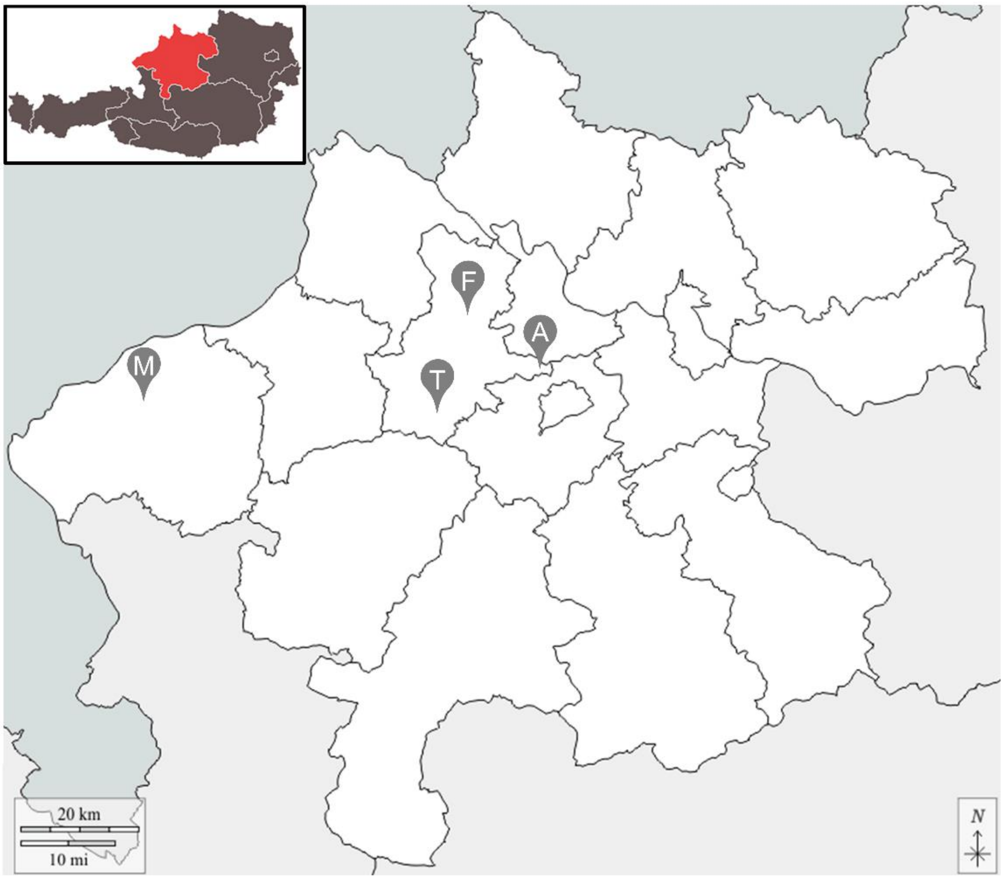

Supplementary Figure S1. Production sites of soybean seeds of various lots. Each seed lot was produced from plants of a certain cultivar grown in separate fields within the province of Upper Austria whose position in the Austrian federation is depicted in red by the inset. Letter indicates field locations in Upper Austria for the cultivars 'Abelina' (M, Moosbach), 'Amadea' (A, Ansfelden), 'Amandine'-organic (T, Thening), and 'Cordoba' (F, Feldkirchen).

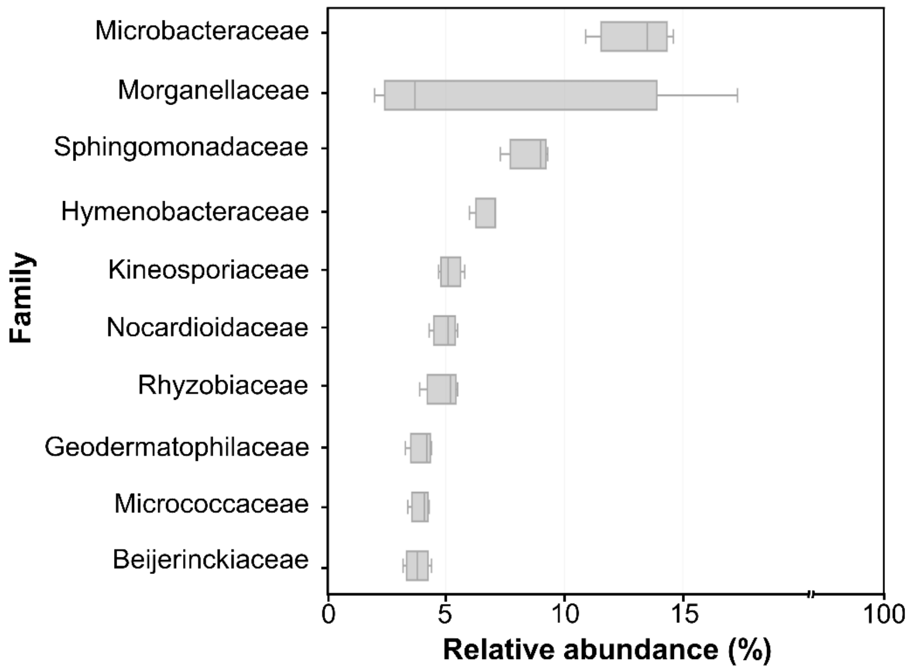

Supplementary Figure S2. Relative abundance of the top 10 bacterial families found on seed coats, in cotyledons and embryonic axes of dry soybean seeds of four cultivars. For an exhaustive lists of taxonomic assignment of all detected amplicon sequence variants in dissected seed compartments refer to Supplementary Table S4.

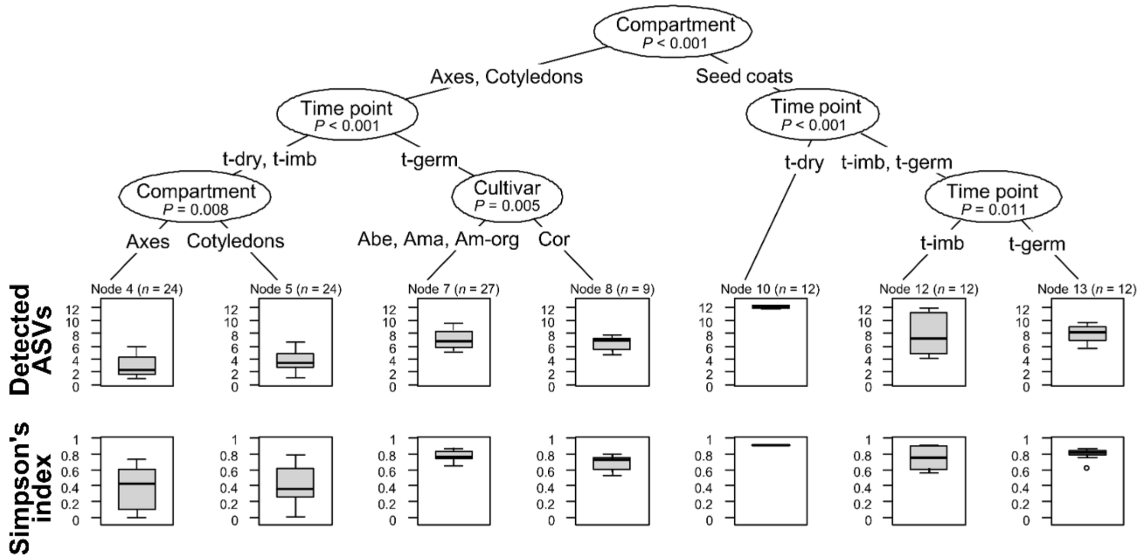

Supplementary Figure S3. Decision tree of bacterial α-diversity based on recursive partitioning analysis. The factors are i) 'seed compartment': embryonic axes (axes), cotyledons, and seed coats; ii) 'time of germination progress': dry seeds (t-dry), imbibed seeds before any had germinated (t-imb), and germinated seeds 5 days after imbibition started (t-germ); iii) soybean 'cultivar': Abelina (Abe), Amadea (Ama), Amadine organic (Am-org), and Cordoba (Cor). Richness, as represented by the number of recovered amplicon sequence variants (ASVs), and evenness, calculated as Simpson's index, are shown as boxplots with outliers denoted by open circles. For each factor, data refer to three replicates of 30 seeds.

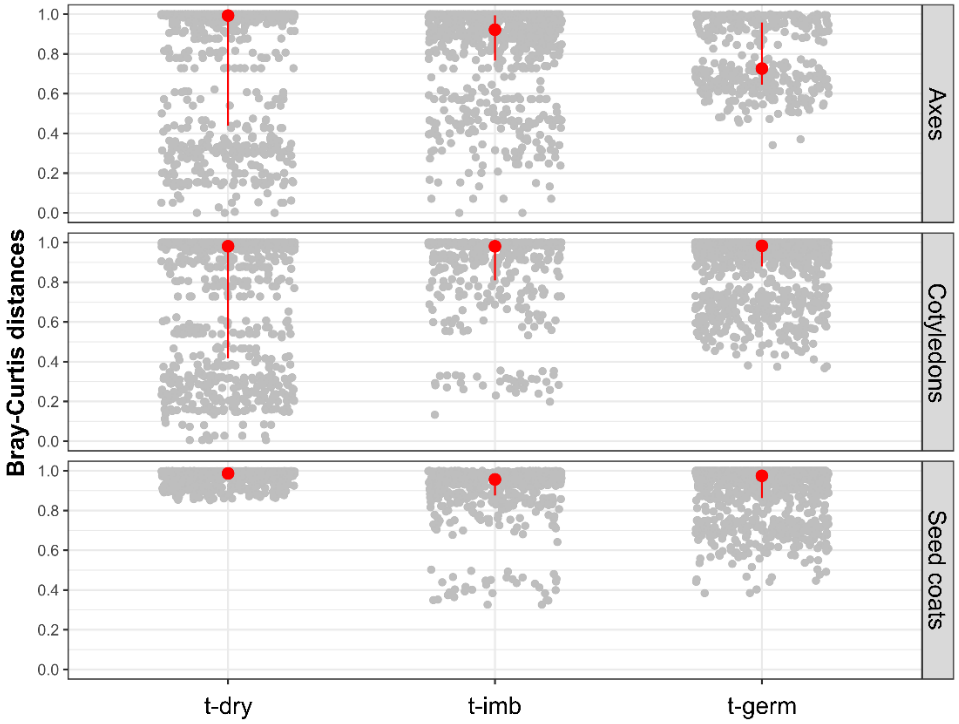

Supplementary Figure S4. Bacterial community changes in each seed compartment within every time point during the progress of soybean germination, viewed through a Jitter plot of Bray-Curtis distances (n = 3 replicates of 30 seeds each). For each seed compartment at a certain time point of germination), summary statistics of the median and inter quartile range were overlayed on the circles considering all four cultivars together. t-dry, dry seeds; t-imb, imbibed seeds before any had germinated; t-germ, germinated seeds 5 days after imbibition started. The term “axes” designates embryonic axes (at t-dry and t-imb) and seedling axes (at t-germ) comprising protruded radicle and nonemerged plumule.

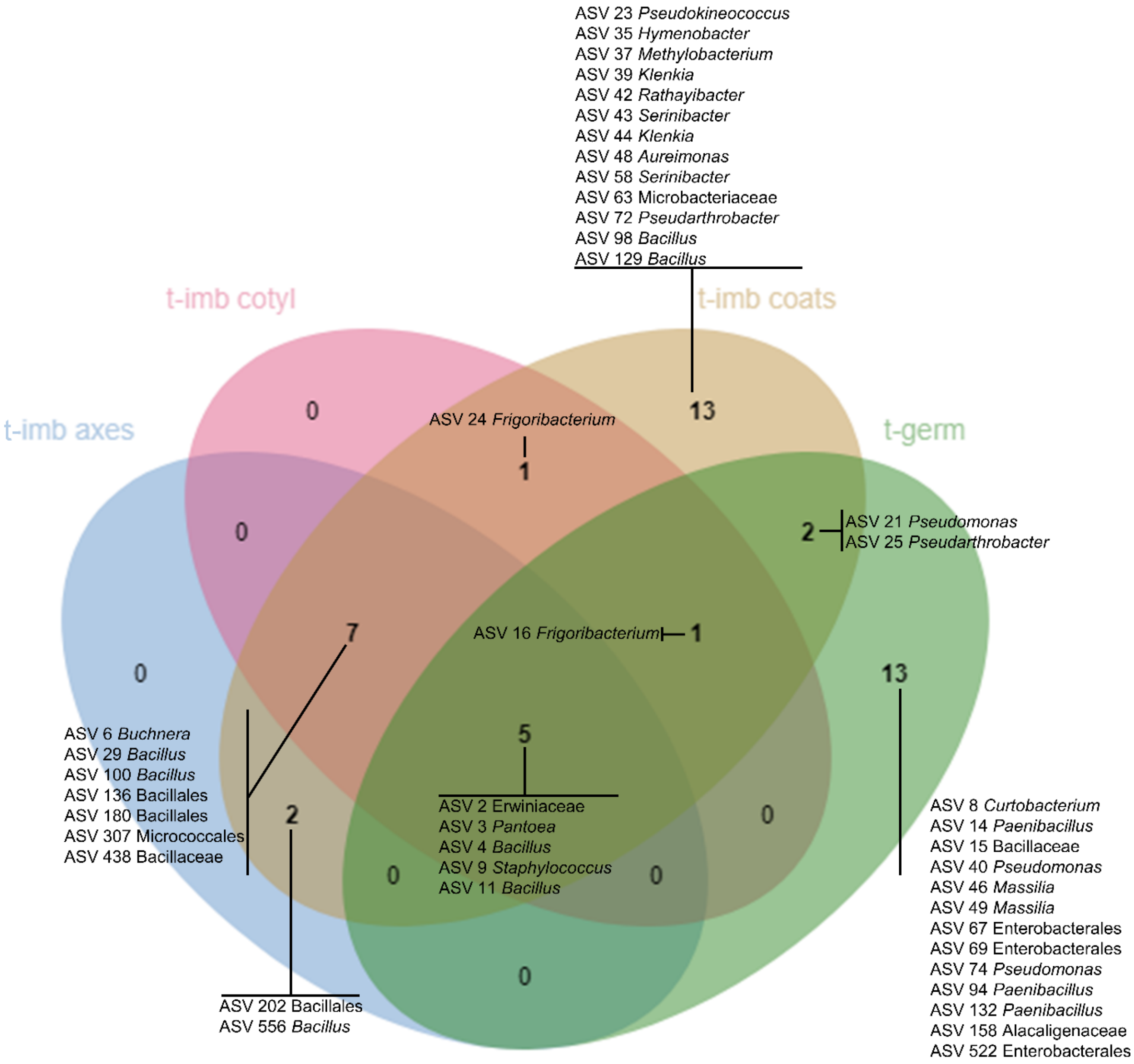

Supplementary Figure S5. Venn diagram showing the core bacterial microbiome shared across four soybean cultivars. For filtering, core amplicon sequence variants (ASVs) with 67% prevalence and 1% threshold were selected for seed compartments (embryonic axes (axes), cotyledons (cotyl), and seed coats (coats)) of imbibed seeds before any had germinated (t-imb) and germinated seeds 5 days after imbibition started (t-germ).

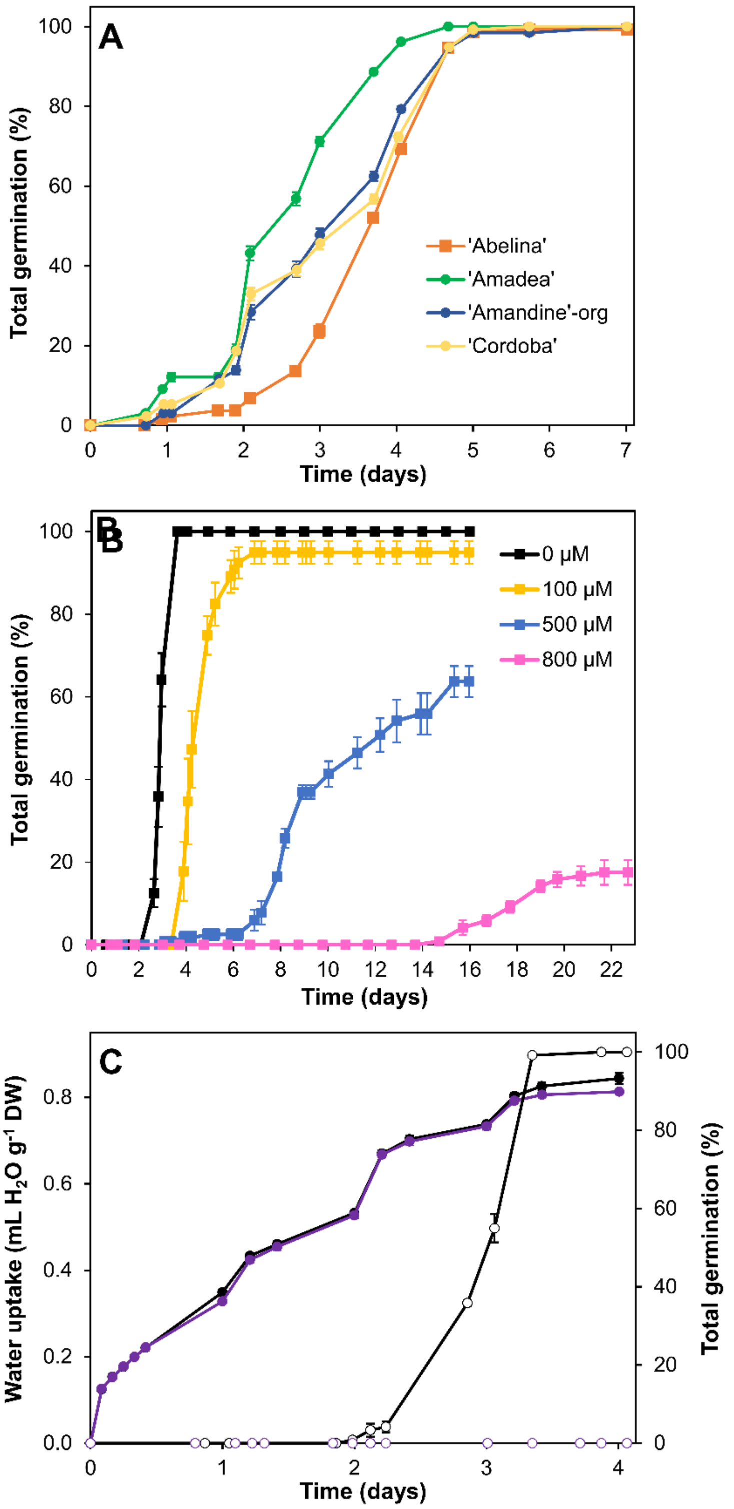

Supplementary Figure S6. Influence of low temperature and exogenous abscisic acid (ABA) on germination and water uptake of soybean seeds. (A) Kinetics of seed germination of four soybean cultivars (key on the right) under controlled fluctuating temperature (10.0 ± 3.5°C). (B) Effect of ABA concentrations (key on the right) on the kinetic of 'Abelina' seed germination at 20 °C in the dark. (C) Effect of 0 µM (control, black curve) and 800 µM ABA (purple curve) on water uptake, measured as increased water volume on a seed dry weight (DW) basis at 20 °C in the dark. Closed and open symbols refer to water uptake and total germination, respectively. Data are means ± SE (*n* = 4 replicates of 30 seeds each).

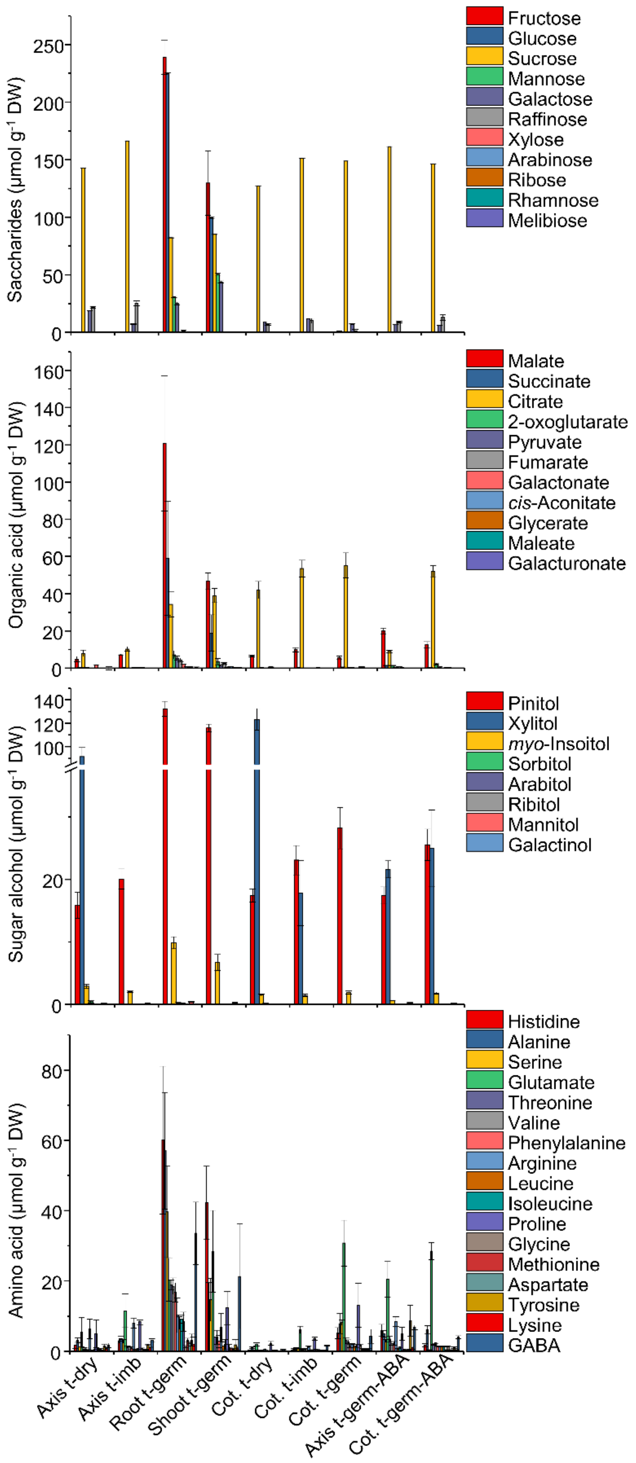

Supplementary Figure S7. Metabolite changes during seed germination and influence of 800 µM abscisic acid (ABA) on the soybean cultivar Abelina. Seeds were analyzed before imbibition (t-dry), before any had germinated (t-imb), and 5 days after imbibition started, when all non-ABA-treated seeds had germinated (t-germ). Targeted metabolites were measured after excising cotyledons (Cot.) and embryonic axes (Axis), which were further split at t-germ into the protruded radicle (root) and non-emerged plumule (shoot), except in ABA-treated seeds (t-germ-ABA) that did not germinate. Data are means ± SD (*n* = 5 replicates of 30 seeds each) normalized on a seed dry weight (DW) basis.

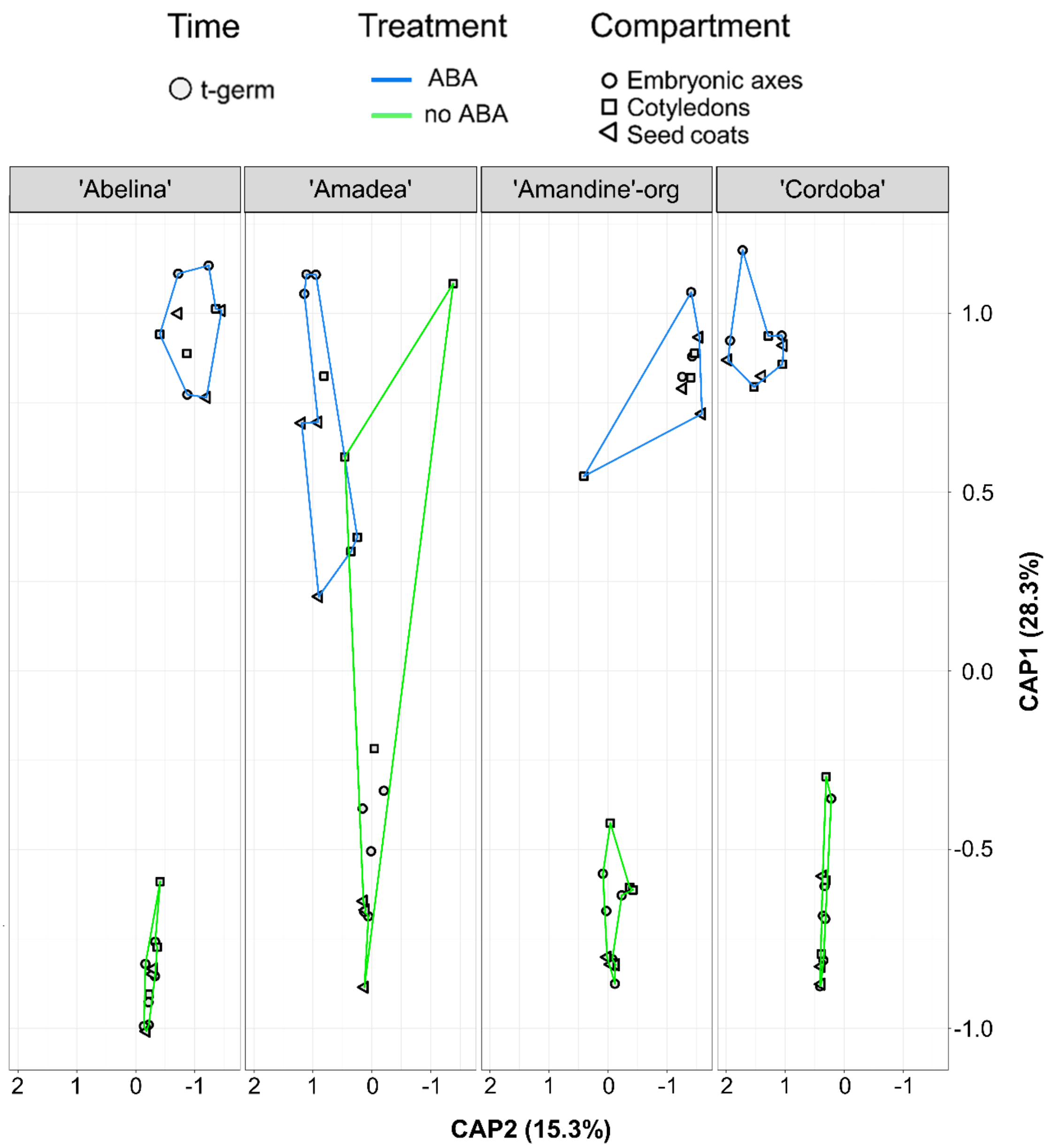

Supplementary Figure S8. Effect of exposure to abscisic acid (ABA) on the structure of bacterial microbiomes of non-germinated soybean seeds 5 days after imbibition with 800 μM ABA started (t-germ-ABA) relative to seeds germinated without exogenous ABA for 5 days (t-germ). Constrained analysis of principal coordinates (CAP) performed on Bray-Curtis dissimilarities considered ABA 'treatment', 'seed compartment', and 'time of germination progress' as constraining factors. CAP ordinations are shown for each cultivar separately ('org' denotes the cultivar grown under organic fertilization regime) for embryonic/seedling axes (circles), cotyledons (squares), and seed coats (triangles). The proportion of variance explained by constrained eigenvalues is reported along the figure axes.

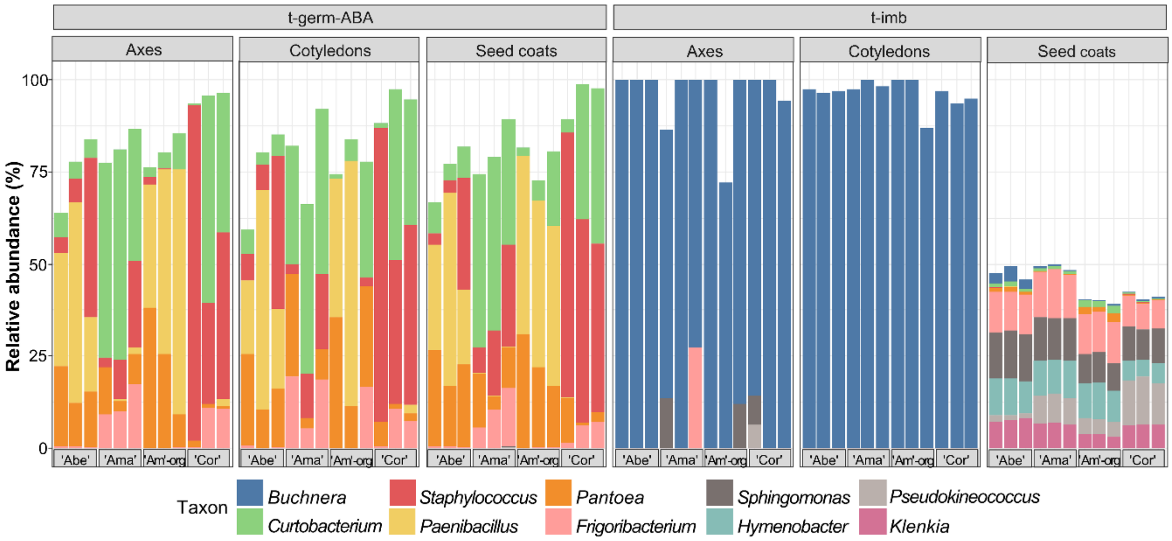

Supplementary Figure S9. Relative abundance of the top 10 bacterial indicator genera influencing the structure of microbiomes of non-germinated seeds after imbibition either in presence or absence (control) of 800 µM exogenous ABA. Vertical bars represent replicates (*n* = 3 of 30 seeds each) ordered by i) 'treatment', ABA (t-germ-ABA) or no ABA (t-imb); ii) 'seed compartment', embryonic axes (axes), cotyledons, and seed coats; iii) 'cultivar': Abelina (Abe), Amadea (Ama), Amadine organic (Am-org), and Cordoba (Cor). Indicator genera are denoted by distinctive colors (see key).

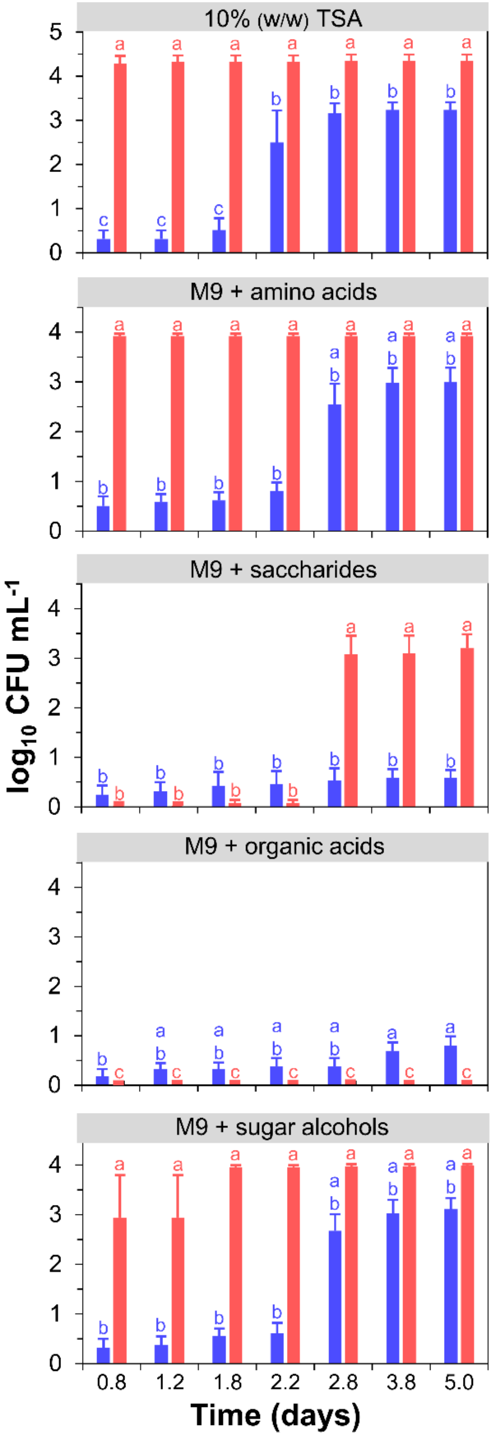

Supplementary Figure S10. Changes over time in the number of colony-forming units (CFU) of selected strains on tryptic soy agar (TSA) and M9 minimal salts-based media supplemented with the most abundant metabolites targeted in each biochemical class (gray headings) at concentrations detected in the seedling axes of germinated seeds 5 days after imbibition started (t-germ). Data are means ± SE (n = 4 for each strain) for *Rhodococcus fascians* (ASV 88, blue) and *Pantoea agglomerans* (ASV 3, red), and different letters denote significant differences (non-parametric Kruskal-Wallis rank tests followed by the Bonferroni correction; P value < 0.05). For comparison with media tailored to the metabolite composition of cotyledons of t-germ seeds refer to Figure 6B.
